# FANCD2 knockdown suppresses malignant phenotypes and is associated with caspase/PARP-mediated apoptosis in NCI-H2052 pleural mesothelioma cells

**DOI:** 10.3389/fgene.2026.1926600

**Published:** 2026-09-23

**Authors:** YingFang Xiang, Pu Su, RuiYing Zhang, Dan Zhao, ZhengLiang Li, WenZhong Yang, ChangCheng Lei, HongYun Chen, Jing Zhang, Wei Xiong

**Affiliations:** 1 College of Basic Medical Sciences, Health Science Center, Dali University, Dali, Yunnan, China; 2 Department of Respiratory and Critical Care Medicine, The First Affiliated Hospital of Dali University, Dali, Yunnan, China; 3 Department of Radiology, The People's Hospital of Lincang City, Lincang, Yunnan, China; 4 Yunnan Provincial Key Laboratory of Entomological Biopharmaceutical R&D, College of Pharmacy, Dali University, Dali, Yunnan, China; 5 Department of Radiology, The First Affiliated Hospital of Dali University, Dali, Yunnan, China; 6 Department of Thoracic Surgery, The First Affiliated Hospital of Kunming Medical University, Kunming, Yunnan, China; 7 College of Public Health, Dali University, Dali, Yunnan, China

**Keywords:** apoptosis, bioinformatics, fanconi anemia complementation group D2, pleural mesothelioma, prognosis

## Abstract

**Background:**

Malignant pleural mesothelioma (PM) is a highly aggressive thoracic malignancy with an extremely poor prognosis and limited effective therapeutic strategies, representing a critical unmet clinical need. Fanconi anemia complementation group D2 (FANCD2) is a critical effector of the cellular DNA damage response system and plays a central role in maintaining genomic integrity. While the genoprotective functions of *FANCD2* have been extensively characterized, its specific biological roles and molecular regulatory mechanisms in the malignant progression of PM remain poorly understood. The current study aimed to evaluate FANCD2 expression and its clinical associations in PM and to examine the effects of transient FANCD2 knockdown in NCI-H2052 cells.

**Methods:**

Comprehensive bioinformatics analyses were performed based on public transcriptomic data from The Cancer Genome Atlas (TCGA) and Gene Expression Omnibus (GEO) databases to profile *FANCD2* expression patterns and evaluate its clinical prognostic value in PM. Differential expression analysis was applied to confirm the aberrant upregulation of *FANCD2* in PM tissues, and the associations of *FANCD2* expression levels with tumor pathological staging and patient clinical prognosis were systematically assessed. Western blotting was further performed to evaluate the protein expression status of FANCD2 in clinical PM specimens and NCI-H2052 cells, with normal tissues and corresponding normal cells included as control groups. Transient *FANCD2* knockdown was achieved in NCI-H2052 cells using two independent small interfering RNAs (siRNAs). A series of functional experiments were conducted to determine the effects of *FANCD2* silencing on the proliferation, wound closure, and apoptosis of NCI-H2052 cells. For mechanistic exploration, the expression levels of core apoptotic proteins were detected to evaluate the association between *FANCD2* depletion and Caspase/PARP-related apoptotic signaling. In addition, tumor immune infiltration analysis was performed to investigate the correlation between FANCD2 expression and M2 macrophage infiltration within the PM tumor microenvironment (TME). FANCD2 rescue and Z-VAD-FMK inhibition experiments were performed to further evaluate the involvement of caspase-dependent apoptosis.

**Results:**

Bioinformatics results demonstrated that *FANCD2* was significantly upregulated in PM tissues relative to normal control samples. Higher FANCD2 expression was associated with tumor T stage and unfavorable survival outcomes in the exploratory TCGA-MESO analysis. Consistently, Western blotting confirmed markedly increased FANCD2 protein levels in both clinical PM tissues and NCI-H2052 cells. Functional assays in NCI-H2052 cells revealed that siRNA-mediated *FANCD2* knockdown significantly suppressed cell proliferation and reduced wound-closure capacity of NCI-H2052 cells, whereas it significantly increased apoptosis. Mechanistic investigations further found that *FANCD2* silencing was associated with increased levels of caspase/PARP-related apoptotic markers, as evidenced by the markedly enhanced protein levels of cleaved caspase-3, cleaved caspase-8, cleaved caspase-9, and cleaved PARP. FANCD2 restoration and pharmacological caspase inhibition partially reversed FANCD2 knockdown-induced growth suppression and apoptosis, further supporting the involvement of caspase-dependent apoptotic signaling. Moreover, TME immune correlation analysis indicated that high FANCD2 expression was positively associated with M2 macrophage infiltration in PM tumors. These findings represent exploratory correlations derived from computational immune-cell estimation.

**Conclusion:**

In summary, FANCD2 may contribute to PM progression by supporting malignant phenotypes and limiting apoptosis. FANCD2 restoration and caspase inhibition partially reversed the growth suppression and apoptosis induced by FANCD2 knockdown, supporting the involvement of caspase-dependent apoptotic signaling. In addition, FANCD2 expression was positively associated with the computationally estimated proportion of M2 macrophages, suggesting that FANCD2 may represent an exploratory prognostic marker and a candidate therapeutic target for further investigation in PM.

## 1 Introduction

Pleural mesothelioma (PM) is an uncommon yet treatment-resistant neoplasm originating from the mesothelial lining of the pleural cavity (Janes et al., 2021). Histologically, PM is categorized into epithelioid, biphasic, and sarcomatoid subtypes, with the epithelioid type comprising approximately 80% of cases and associated with a relatively more favorable prognosis (Galateau-Salle et al., 2016). Diagnosis relies on clinical presentation, imaging studies, and pathological confirmation, for which pleural biopsy remains the gold standard for pathological diagnosis (Carbone et al., 2019). Although progressive dyspnea secondary to pleural effusion is the most common clinical presentation, PM is often diagnosed at an advanced stage due to considerable diagnostic difficulties (Harris et al., 2019). PM is largely refractory to a range of multimodal treatments, including surgical resection, cisplatin-pemetrexed chemotherapy, and radiotherapy. Consequently, the median survival is 9–12 months, with a 5-year survival rate of roughly 5% (Meyerhoff et al., 2015).

Molecular studies have shown that tumor suppressor genes and epigenetic regulators, including BRCA1-associated protein 1 (BAP1), cyclin-dependent kinase inhibitor 2A (CDKN2A), cyclin-dependent kinase inhibitor 2B (CDKN2B), neurofibromin 2 (NF2), methylthioadenosine phosphorylase (MTAP), and tumor protein P53 (TP53), are commonly altered in PM, with recurrent genomic alterations reported in these genes (Shao et al., 2020; Hiltbrunner et al., 2022). Moreover, pathogenic alterations involving DNA repair-related genes, including BAP1, CDKN2A, ataxia telangiectasia mutated (ATM), and Fanconi anemia complementation group D2 (FANCD2), have been reported in PM (Belcaid et al., 2023). The genetic landscape of PM is characterized by recurrent alterations in tumor suppressor and genome-maintenance genes, including BAP1, CDKN2A, NF2, TP53, and ATM. These alterations may impair DNA-damage sensing, cell-cycle checkpoint control, and DNA repair. In addition, asbestos exposure induces oxidative stress, DNA strand breaks, and replication stress, thereby increasing the dependence of PM cells on functional DNA-damage-response pathways. As a central component of the Fanconi anemia pathway, FANCD2 contributes to the repair of DNA interstrand crosslinks, stabilization of stalled replication forks, and maintenance of genomic integrity. Therefore, aberrant FANCD2 expression may enhance the ability of PM cells to tolerate persistent DNA damage and replication stress. Fanconi anemia (FA) is a rare inherited disorder characterized by bone marrow failure, congenital abnormalities, and increased cancer susceptibility (Nepal et al., 2017; Sala-Trepat et al., 2000). The FA signaling pathway is a central regulator of the DNA damage and replication stress response. The monoubiquitination of the FANCD2-FANCI core complex is a hallmark event required for pathway activation (Li et al., 2020). The FANCD2 gene, located on chromosome 3p25.3 and comprising 45 exons, is a pivotal component of this pathway. Defects in the FA pathway, in which FANCD2 serves as a key effector, can contribute to genomic instability and tumorigenesis. Accumulating evidence indicates that FANCD2 is overexpressed in hepatitis B virus (HBV)-related hepatocellular carcinoma (Tang et al., 2023). FANCD2 has also been reported to promote proliferation, invasion, and migration in lung adenocarcinoma cells (Xie et al., 2024). It is also highly expressed in medulloblastoma (SHH-MB subtype) (Zhou et al., 2024), endometrial carcinoma and osteosarcoma (Zheng et al., 2022; Li and Liu, 2023). Nevertheless, the expression profile and clinical relevance of *FANCD2* in PM remain unclear. Therefore, this study aimed to characterize FANCD2 expression and its clinical associations in PM and to examine the effects of transient FANCD2 knockdown on cell viability, colony formation, wound-closure capacity, cell-cycle distribution, and apoptosis in NCI-H2052 cells.

## 2 Materials and methods

### 2.1 Bioinformatics analysis

To assess FANCD2 mRNA expression across different malignancies, the Tumor Immune Estimation Resource (TIMER; cistrome.shinyapps.io/timer) database was initially interrogated. Mesothelioma (MESO), lung adenocarcinoma (LUAD), and lung squamous carcinoma (LUSC) datasets were obtained from the UCSC Xena platform (http://xena.ucsc.edu/welcome-to-ucsc-xena/), and MESO-related datasets were further supplemented with independent cohorts from the Gene Expression Omnibus (GEO) database (https://www.ncbi.nlm.nih.gov/geo/). Expression matrices were extracted using Perl software (version 5.40.2.1), and Ensembl IDs were converted to gene IDs. FANCD2 expression levels were analyzed using the R version 4.3.1 with the limma, ggpubr, clusterProfiler, enrichplot, and pheatmap packages, and differential expression analyses were performed between tumor and reference samples. Because the TCGA-MESO cohort lacks matched normal pleural mesothelial tissues, 108 non-tumor lung samples from the normal tissue subsets of the TCGA-LUAD and TCGA-LUSC cohorts were used as an exploratory reference. The TCGA and GEO datasets were analyzed independently rather than merged across platforms; therefore, cross-platform batch effect correction was not required. Considering potential tissue-of-origin differences, *FANCD2* expression patterns were further evaluated in independent GEO cohorts containing normal pleural mesothelial samples or non-tumor pleural samples. The sample sources and numbers used in the bioinformatics analyses are summarized in Supplementary Table 1.

### 2.2 Clinicopathological characteristics analysis of FANCD2 expression

Clinicopathological data for patients in the TCGA-MESO cohort were obtained from UCSC Xena and matched with gene expression data using sample identifiers. Based on the median expression of FANCD2, PM patients were stratified into high- and low-FANCD2 expression cohorts (minimum: 0.9979, median: 2.6210, maximum: 4.7142). To identify clinical correlates of FANCD2 expression, we conducted univariate and multivariate analyses using the epicalc and cdgsr packages in R4.3.1 software. These analyses evaluated its relationship with factors such as age, gender, AJCC staging, and histological subtype. Associations between FANCD2 expression group and clinicopathological characteristics were evaluated using Pearson’s chi-square test or Fisher’s exact test, as appropriate. To further investigate the association between FANCD2 expression and clinicopathological characteristics in MESO, transcriptomic and corresponding clinical data from TCGA were analyzed using the UALCAN web portal (Chandrashekar et al., 2017; Chandrashekar et al., 2022). FANCD2 mRNA expression was expressed as transcripts per million (TPM). MESO samples were stratified according to pathological stage (stages I–IV), sex, age (21–40, 41–60, 61–80, and 81–100 years), histological subtype (biphasic, epithelioid, diffuse malignant, and sarcomatoid mesothelioma), lymph-node metastasis status (N0–N3), and TP53 mutation status. Differences in FANCD2 expression between subgroups were assessed using the statistical analysis implemented in UALCAN. Pairwise comparisons were performed using an unpaired two-sample Student’s t-test. A two-sided P value <0.05 was considered statistically significant.

### 2.3 Survival analysis of patients with PM

Patients with available overall survival (OS) data in the TCGA-MESO cohort were included in the survival analysis. Patients were divided into high- and low-FANCD2 expression groups according to the median FANCD2 expression level. Kaplan–Meier survival curves were generated, and differences between groups were evaluated using the log-rank test. Overall survival (OS) and disease-free survival (DFS) analyses were additionally performed using GEPIA2 official website (http://gepia2.cancer-pku.cn/#index) with the TCGA-MESO dataset, using the median FANCD2 expression level as the group cutoff and displaying 95% confidence intervals (CI).

To further evaluate the association between FANCD2 expression and OS, univariate and multivariable Cox proportional hazards regression analyses were performed. Age was categorized as ≥60 versus <60 years. FANCD2 expression was dichotomized at the median. Sex, AJCC stage, and histological subtype were included as categorical variables. Female sex, AJCC stage I, epithelioid histology, and low FANCD2 expression were used as the corresponding reference categories. To reduce model overparameterization and avoid redundant adjustment for correlated staging variables, the multivariable Cox model included only FANCD2 expression, age group, sex, AJCC stage, and histological subtype. T, N, and M categories were not entered separately into the multivariable model together with AJCC stage. TX, NX, and MX were regarded as unknown or unassessable categories rather than biological stages and were not assigned separate hazard-ratio estimates. Hazard ratios (HRs), 95% CIs, and two-sided P values were reported. The proportional hazards assumption was assessed using Schoenfeld residuals.

### 2.4 Immune infiltration analysis

The ESTIMATE algorithm was used to calculate the StromalScore, ImmuneScore, and ESTIMATEScore for each TCGA-MESO sample and to evaluate the association between FANCD2 expression and the tumor microenvironment. The relative abundance of 22 tumor-infiltrating immune-cell types was estimated using the CIBERSORT algorithm with the LM22 signature matrix and 1,000 permutations. Only samples with a CIBERSORT output *P* value <0.05 were included in subsequent analyses. Differences in immune-cell infiltration between the high-expression and low-expression FANCD2 groups were assessed, and correlations between FANCD2 expression and immune-cell proportions were evaluated using Spearman correlation analysis. Benjamini–Hochberg correction was applied across the tested immune-cell types to control the false-discovery rate. Tumor purity was estimated using the ESTIMATE framework but was not included as a covariate in the correlation analyses. Therefore, residual confounding by tumor purity and stromal content could not be excluded, and all immune-infiltration associations were considered exploratory.

### 2.5 Gene set enrichment analysis (GSEA)

Gene Ontology (GO) or Kyoto Encyclopedia of Genes and Genomes (KEGG) enrichment analyses were conducted to investigate biological processes and signaling pathways associated with FANCD2 expression in PM (Szklarczyk et al., 2021). For GSEA, the normalized gene-level RNA-sequencing expression matrix of the TCGA-MESO cohort was obtained from the UCSC Xena platform. Ensembl gene identifiers were mapped to official gene symbols using the corresponding GENCODE annotation. Genes that could not be mapped were removed, and duplicate gene symbols generated during identifier conversion were collapsed by retaining their mean expression value. Because the TCGA-MESO data were derived from a single RNA-sequencing platform and were analyzed independently, no additional cross-platform batch-effect correction was performed. Expression values were log2-transformed as log2 (x+1) when necessary. Samples were divided into high- and low-FANCD2 expression groups according to the median FANCD2 expression level. Differential expression analysis between the two groups was performed using the limma package in R, and all genes were ranked according to their log2 fold-change values. Preranked GSEA was performed using the clusterProfiler package in R software (4.3.1) with gene sets obtained from the Molecular Signatures Database (MSigDB). The minimum and maximum gene-set sizes were set to 10 and 500, respectively, and 1,000 permutations were performed. P values were adjusted using the Benjamini–Hochberg method. Gene sets with an adjusted *P* value <0.05 and an absolute normalized enrichment score (NES) > 1 were considered significantly enriched. GSEA results were visualized using the enrichplot package. Additionally, using the TCGA mesothelioma dataset, the R software (4.3.1) “pheatmap” package was employed to filter out genes that were highly correlated with *FANCD2* expression in PM. Functional enrichment analysis was conducted in R using packages such as clusterProfiler and enrichplot to interpret the co-expressed genes in the context of GO terms and KEGG pathways.

### 2.6 Tissue sample collection

Ten PM tissue specimens and their paired adjacent non-tumor tissue specimens were collected from ten patients who underwent surgery and received a pathological diagnosis of PM in the Department of Thoracic Surgery at the First Affiliated Hospital of Kunming Medical University between September 2022 and October 2024. The inclusion criteria were as follows: (1) no antitumor treatment before surgery and (2) complete clinical records and available tissue specimens. Patients with another concurrent malignant tumor or non-primary PM were excluded. The cohort comprised six male and four female patients, with a mean age of 57 years and an age range of 45–65 years. Six patients had stage I-II disease and four had stage III disease. Written informed consent was obtained from all patients. All procedures were performed in accordance with the ethical standards of the Medical Ethics Review Committee of the First Affiliated Hospital of Dali University, which approved the study under approval number KYLL-202203.

### 2.7 Cell lines and cell culture

The human normal pleural mesothelial cell line MeT-5A was obtained from Shanghai Qingqi Biotechnology Development Co., Ltd., and the human PM cell lines (MSTO-211H, NCI-H28, NCI-H2052, NCI-H2452) were obtained from the Shanghai Cell Bank, Chinese Academy of Sciences, respectively. All cell lines were authenticated by short tandem repeat (STR) profiling and confirmed to be mycoplasma-free. Human MeT-5A cells were cultured in medium 199 (Thermo Fisher Scientific, USA) containing Earle’s balanced salt solution (BSS), 0.75 mmol/L L-glutamine, and 1.25 g/L sodium bicarbonate, supplemented with 3.3 nmol/L epidermal growth factor, 400 nmol/L hydrocortisone, 870 nmol/L insulin, 20 mmol/L 4-(2-hydroxyethyl)-1-piperazineethanesulfonic acid (HEPES) buffer, and 10% (v/v) fetal bovine serum (FBS; E01030, EvaCell). All human PM cell lines employed in this study were cultivated *in vitro* utilizing RPMI-1640 medium supplemented with 10% FBS (E01030, EvaCell) and 1% antibiotic mixture containing 100 U/mL penicillin and 100 μg/mL streptomycin (E3102, EvaCell, Hong Kong, China) in a 37 °C, 5% CO2 incubator. The medium was replaced every 2 days. When cell density reached 80%–90%, cells were digested with trypsin solution for passaging or seeding into new culture plates.

### 2.8 Cell transfection

Human NCI-H2052 cells were seeded in 6-well plates at 2.5 × 10^5^ cells/well with antibiotic-free RPMI 1640 medium and cultured for 20–24 h to 45%–55% confluency. Three siRNA groups were established: si-NC, si-FANCD2-1, and si-FANCD2-2. The siRNAs were purchased from GenePharma Co., Ltd. (Shanghai, China; Supplementary Table 2). For each group, 5 μL of 20 μM siRNA was diluted in 250 μL Opti-MEM, and 4 μL Lipofectamine 3,000 was diluted in another 250 μL Opti-MEM. The two solutions were gently mixed and incubated at room temperature for 18 min to form complexes. After aspirating the original medium, 1.5 mL fresh Opti-MEM was added to each well, followed by 500 μL of the complexes. After incubation at 37 °C, 5% CO_2_ for 5 h, the medium was replaced with 2 mL antibiotic-free RPMI 1640 medium. Cells were cultured for another 48 h post-transfection and harvested for Western blotting and qRT-PCR to evaluate *FANCD2* silencing efficiency.

### 2.9 Quantitative reverse-transcription PCR (qRT-PCR)

Total RNA was extracted from tissue specimens, MeT-5A cells, and PM cell lines using the RNAsimple Total RNA Extraction Kit (TIANGEN Biotech Co., Ltd., Beijing, China; Cat. No. DP419), and cDNA was synthesized following the HiScript II RT SuperMix for qPCR (+gDNA wiper) protocol. qRT-PCR was carried out on a real-time fluorescence PCR instrument (Aipubio Biotechnology Co., Ltd.) to analyze cDNA templates from MeT-5A cells and different PM cell lines, using the Servicebio 2× Universal Blue SYBR Green qPCR Master Mix. All procedures were performed following the manufacturer’s protocol. Relative quantification was conducted using the 2^−ΔΔCt^ method with β-actin as the reference gene. The primer sequences are included in Supplementary Table 2. All experiments were carried out with technical triplicates and repeated independently three times, with data expressed as the mean.

### 2.10 Western blotting

For tissue samples, frozen tissue specimens were homogenized in radio immunoprecipitation assay (RIPA) lysis buffer containing protease inhibitors, followed by centrifugation to collect total protein. For cell samples, cells were harvested 48 h after treatment and lysed in 150 μL of precooled RIPA lysis buffer on ice for 30 min. After centrifugation, the supernatants were collected, and protein concentrations were determined using the bicinchoninic acid (BCA) method. Equal amounts of protein were denatured and separated by sodium dodecyl sulfate-polyacrylamide gel electrophoresis (SDS-PAGE) at 80 V for 40 min and 120 V for 90 min. The separated proteins were subsequently transferred onto polyvinylidene fluoride (PVDF) membranes. The membranes were blocked with 5% skimmed milk and incubated overnight at 4 °C with the corresponding primary antibodies listed in Supplementary Table 3. After washing, the membranes were incubated with horseradish peroxidase (HRP)-conjugated secondary antibodies at room temperature for 50 min. Protein bands were visualized using enhanced chemiluminescence reagents. Band intensities were quantified using ImageJ software. Each target-protein band was normalized to its corresponding loading control, and the si-NC group was normalized to 1.0. Densitometric analysis was independently performed for three biological experiments, and representative bands were selected after completion of the quantitative analysis. Densitometric values were calculated independently from the original, unsaturated exposures of three biological replicates. The representative Western blot shown in each figure was selected from these biological replicates and was not itself used as the sole basis for statistical analysis.

### 2.11 Cell Counting Kit-8 (CCK-8) assay

Cell viability was evaluated using the Cell Counting Kit-8 (CCK-8; Dojindo, Japan). Briefly, cells were seeded into 96-well plates at a density of 3 × 10^3^ cells per well and cultured under the indicated conditions. At 24, 48, and 96 h, 10 μL of CCK-8 reagent was added to each well, followed by incubation at 37 °C for 2 h. The absorbance was then measured at 450 nm using a microplate reader. All experiments were independently repeated three times.

### 2.12 Colony formation assay

At 24–48 h after siRNA transfection, NCI-H2052 cells were harvested, counted, and replated into 6-well plates at 1,000 cells/well for the colony-formation assay. Colonies were fixed with 4% paraformaldehyde (30 min), then stained with 0.5% crystal violet (30 min), washed with PBS, and imaged. Colonies were quantified using ImageJ. The experiment was independently repeated three times, and the average value was calculated.

### 2.13 Wound healing assay

Cells were grown to >95% confluence in 6-well plates, and three scratches were made per well with a sterile pipette tip. Images were captured immediately after scratching (0 h) and at 48 h. The wound-closure rate was quantified as the proportion of the initial scratch area that was filled by cells over 48 h. The experiment was independently repeated three times.

### 2.14 Cell cycle distribution analysis

Following a 48-h culture, cells were harvested, fixed in cold 70% ethanol for over 12 h, and stained with PI/RNase A (50 μg/mL and 100 μg/mL, respectively) prior to cell cycle analysis on a Beckman A00-1-1102 flow cytometer. The proportions of cells in G0/G1, S, and G2/M phases were quantified by the instrument’s software. The experiment was independently repeated three times.

### 2.15 Cell apoptosis analysis

Transfected NCI-H2052 cells and the corresponding culture supernatants were collected and washed with PBS. The cells were resuspended in 500 μL of precooled 1× binding buffer, followed by the addition of 5 μL of Annexin V-FITC and 5 μL of propidium iodide. After incubation for 8–10 min at room temperature in the dark, the samples were immediately analyzed using a CyFlow Cube 8 flow cytometer. Data were analyzed using FCS Express V3 and FlowJo version 10.8.1. The experiment was independently repeated three times.

### 2.16 FANCD2 rescue and caspase inhibition experiments

To further determine whether the effects of FANCD2 depletion involved caspase-dependent apoptotic signaling, NCI-H2052 cells were subjected to FANCD2 rescue and pharmacological caspase-inhibition experiments. The si-FANCD2-2 sequence, which showed the higher knockdown efficiency, was used for subsequent rescue experiments. For FANCD2 rescue, cells were transfected with a FANCD2-overexpression (OE) plasmid (FANCD2 [NM_033084] Human Untagged Clone, Cat. No. SC305556; OriGene Technologies, Inc., Rockville, MD, USA) or empty plasmid (vector) at 2.5 μg/well using Lipofectamine 3,000 according to the manufacturer’s instructions. The FANCD2 expression construct was select the applicable description: an siRNA-resistant FANCD2 construct containing silent substitutions within the siFANCD2 target sequence/a FANCD2 coding-sequence construct lacking the 3′UTR, whereas siFANCD2 targeted the endogenous FANCD2 3′UTR, allowing restoration of exogenous FANCD2 expression in FANCD2-silenced cells. The corresponding empty vector was used as the vector control. For caspase inhibition, cells were treated with the pan-caspase inhibitor Z-VAD-FMK (Selleck Chemicals, Houston, TX, USA; Cat. No. S7032) at a final concentration of 20 μM. Z-VAD-FMK was dissolved in dimethyl sulfoxide (DMSO), and cells were pretreated for 1 h before subsequent experimental treatment and maintained throughout the indicated experimental period. Cells used for Western blotting and apoptosis assays were collected after 48 h of treatment. Cells were divided into five groups: siNC + Vector, siFANCD2-2 + Vector, siFANCD2-2 + FANCD2-OE, siNC + Vector + Z-VAD-FMK, and siFANCD2-2 + Vector + Z-VAD-FMK.

Cell viability was assessed using the CCK-8 assay at 0, 24, 48, and 72 h. Colony formation was evaluated as described above. Apoptosis was assessed by Annexin V-FITC/PI staining followed by flow cytometry, with total apoptosis calculated as the sum of early and late apoptotic cells. Western blotting was performed to determine the levels of FANCD2, cleaved caspase-3, cleaved caspase-8, cleaved caspase-9, and cleaved PARP, with GAPDH used as the loading control. All experiments were independently repeated three times.

### 2.17 Statistical analysis

Statistical analyses were performed using R version 4.3.1 and GraphPad Prism 10.0, together with the statistical procedures implemented in the indicated online databases. All statistical tests were two-sided. For high-dimensional analyses involving simultaneous testing of multiple immune-cell types, co-expressed genes, immune checkpoint-related genes, GO/KEGG terms, or GSEA gene sets, P values were adjusted using the Benjamini–Hochberg procedure to control the false-discovery rate. Adjusted P values <0.05 were considered statistically significant unless otherwise specified. For experimental comparisons involving more than two groups, one-way ANOVA followed by Dunnett’s multiple-comparisons test was used. Time-course CCK-8 data were analyzed using two-way ANOVA followed by Dunnett’s multiple-comparisons test. Paired PM and adjacent non-tumor tissue samples were compared using paired t-tests. Clinicopathological associations were evaluated using Pearson’s chi-square test or Fisher’s exact test, as appropriate, and survival outcomes were analyzed using Kaplan–Meier analysis, log-rank tests, and Cox proportional-hazards regression. Because the clinicopathological and survival analyses were prespecified exploratory analyses, nominal two-sided P values are reported for these individual clinical comparisons, with P < 0.05 considered nominally significant.

## 3 Results

### 3.1 FANCD2 mRNA expression is elevated in PM tissues

TIMER database analysis showed that the expression of FANCD2 in 22 different types of tumor tissues was significantly higher than that in corresponding normal tissues, including bladder cancer (BLCA), breast cancer (BRCA), rectal adenocarcinoma (READ), and gastric adenocarcinoma (STAD), etc (all *P* < 0.05, Figure 1A). Furthermore, we observed elevated *FANCD2* mRNA expression in 86 PM samples compared to 108 normal lung tissue controls from the TCGA database (*P* < 0.001, Figure 1B). This upregulation was consistently validated in three independent GEO datasets (GSE12345, GSE42977, and GSE51024), where *FANCD2* mRNA levels were significantly higher in PM tissues than in normal pleural mesothelial tissues (*P* < 0.01; *P* < 0.001, Figures 1C–E).

**FIGURE 1:**
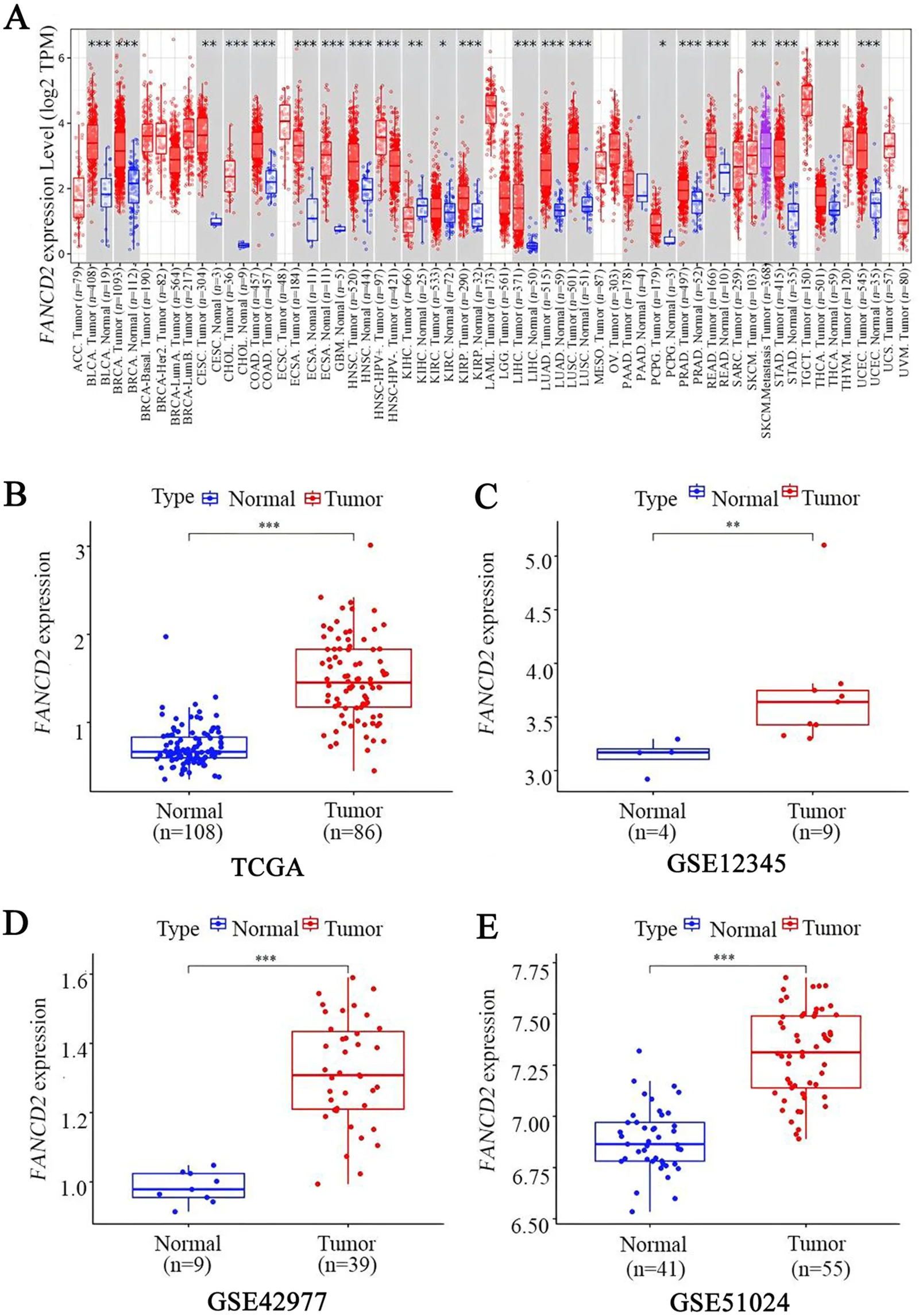
Expression of *FANCD2* gene in normal tissues and tumor tissues. **(A)** Pan-cancer analysis of *FANCD2* gene expression. **(B)** Expression of *FANCD2* mRNA in the TCGA database. **(C)** Expression of *FANCD2* mRNA in the GEO GSE12345 dataset. **(D)** Expression of *FANCD2* mRNA in the GEO GSE42977 dataset. **(E)** Expression of *FANCD2* mRNA in the GEO GSE51024 dataset. *P < 0.05, **P < 0.01, ***P < 0.001.

### 3.2 Association between *FANCD2* mRNA expression and clinicopathological characteristics in PM patients

Clinicopathologic characteristics were compared between low- and high-FANCD2 expression groups in the TCGA-MESO cohort. No significant correlations were found with gender, AJCC stage, N stage, M stage, or histological subtype (P > 0.05), with the exception of a significant association with age and tumor T stage (P < 0.05) (Table 1). Further subgroup analyses showed that FANCD2 mRNA expression was generally not associated with conventional clinicopathological characteristics in MESO (Supplementary Figure S1). FANCD2 expression did not differ significantly among tumors of different pathological stages (Supplementary Figure S1A). Similarly, no significant difference in FANCD2 expression was observed between male and female patients (Supplementary Figure S1B). When patients were stratified according to age, FANCD2 expression showed some intergroup variation; however, most pairwise comparisons were not statistically significant, with only one age-group comparison reaching statistical significance (P < 0.05) (Supplementary Figure S1C). No significant differences in FANCD2 expression were detected among the different histological subtypes of MESO (Supplementary Figure S1D) or among tumors with different lymph-node metastasis statuses (N0–N3) (Supplementary Figure S1E). In addition, FANCD2 expression was comparable between TP53-mutant and TP53-nonmutant tumors (Supplementary Figure S1F).

**TABLE 1:** Correlation between *FANCD2* mRNA expression and clinicopathological parameters in PM patients.

Clinicopathological parameters	*n*	Low-*FANCD2* expression (%)	High-*FANCD2* expression (%)	*χ* ^*2*^	*P*
Age (years)	​	​	​	3.983	**0.046**
<60	33	21 (63.64)	12 (36.36)	​	​
≥60	53	22 (41.51)	31 (58.49)	​	​
Gender	​	​	​	0.307	0.579
Female	16	7 (43.75)	9 (56.25)	​	​
Male	70	36 (51.43)	34 (48.57)	​	​
AJCC stage	​	​	​	5.105	0.164
Stage I	10	4 (40.00)	6 (60.00)	​	​
Stage II	16	11 (68.75)	5 (31.25)	​	​
Stage III	44	18 (40.91)	26 (59.09)	​	​
Stage IV	16	10 (62.50)	6 (37.50)	​	​
T stage	​	​	​	10.288	**0.036**
T1	14	7 (50.00)	7 (50.00)	​	​
T2	26	17 (65.38)	9 (34.62)	​	​
T3	31	10 (32.26)	21 (67.74)	​	​
T4	13	9 (69.23)	4 (30.77)	​	​
TX	2	0 (0.00)	2 (100.00)	​	​
N stage	​	​	​	1.910	0.752
N0	43	21 (48.84)	22 (51.16)	​	​
N1	10	6 (60.00)	4 (40.00)	​	​
N2	26	14 (53.85)	12 (46.15)	​	​
N3	3	1 (33.33)	2 (66.67)	​	​
NX	4	1 (25.00)	3 (75.00)	​	​
M stage	​	​	​	0.370	0.831
M0	56	28 (50.00)	28 (50.00)	​	​
M1	3	2 (66.67)	1 (33.33)	​	​
MX	27	13 (48.15)	14 (51.85)	​	​
Tumor type	​	​	​	2.934	0.231
Biphasic type	23	8 (34.78)	15 (65.22)	​	​
Epithelioid type	61	34 (55.74)	27 (44.26)	​	​
Sarcomatoid type	2	1 (50.00)	1 (50.00)	​	​

Data are presented as *n* (%). Patients were divided into low- and high-FANCD2 expression groups according to the median FANCD2 mRNA expression level. Associations between FANCD2 expression and clinicopathological characteristics were evaluated using Pearson’s chi-square test or Fisher’s exact test, as appropriate. TX, NX, and MX, indicate unknown or unassessable T, N, and M categories, respectively, and were not considered biological stages. A two-sided *P* < 0.05 was considered statistically significant. AJCC, american joint committee on cancer; PM, pleural mesothelioma; FANCD2, Fanconi anemia complementation group D2. Bold values indicate statistically significant differences (P < 0.05).

Collectively, these findings indicate that FANCD2 expression is largely independent of conventional clinicopathological characteristics in the TCGA-MESO cohort. Given the small sample sizes of some subgroups, particularly the youngest and oldest age groups and several histological or nodal-status subgroups, the isolated age-related difference should be interpreted cautiously and requires validation in larger independent cohorts.

### 3.3 *FANCD2* mRNA expression and prognosis in PM patients

High FANCD2 expression was associated with significantly poorer overall survival (OS) and disease-free survival (DFS) in the TCGA-MESO cohort (OS: log-rank P = 0.00015; DFS: log-rank P = 0.028; Figures 2A,B). Among the 86 patients included in the OS analysis, 73 deaths were observed during follow-up. In univariate Cox proportional hazards regression, high FANCD2 expression was associated with an increased mortality hazard compared with low FANCD2 expression (HR = 2.83, 95% CI: 1.72–4.66, P < 0.001). In the multivariable model adjusted for age group (≥60 vs. <60 years), sex, AJCC stage, and histological subtype, high FANCD2 expression remained associated with poorer OS (HR = 3.02, 95% CI: 1.76–5.16, P < 0.001) (Table 2). Using epithelioid mesothelioma as the reference histological subtype, biphasic histology was associated with a higher mortality hazard in both univariate analysis (HR = 1.98, 95% CI: 1.18–3.33, P = 0.010) and multivariable analysis (HR = 2.23, 95% CI: 1.26–3.97, P = 0.006). The hazard-ratio estimate for sarcomatoid histology was imprecise because of the very small number of cases and should therefore be interpreted with caution. Age group, sex, and AJCC stage were not significantly associated with OS in the multivariable model. Given the modest cohort size, sparse clinicopathological subgroups, median-based dichotomization of FANCD2 expression, and lack of an independent survival-validation cohort, these prognostic associations should be considered exploratory and require validation in larger, well-annotated PM cohorts.

**FIGURE 2:**
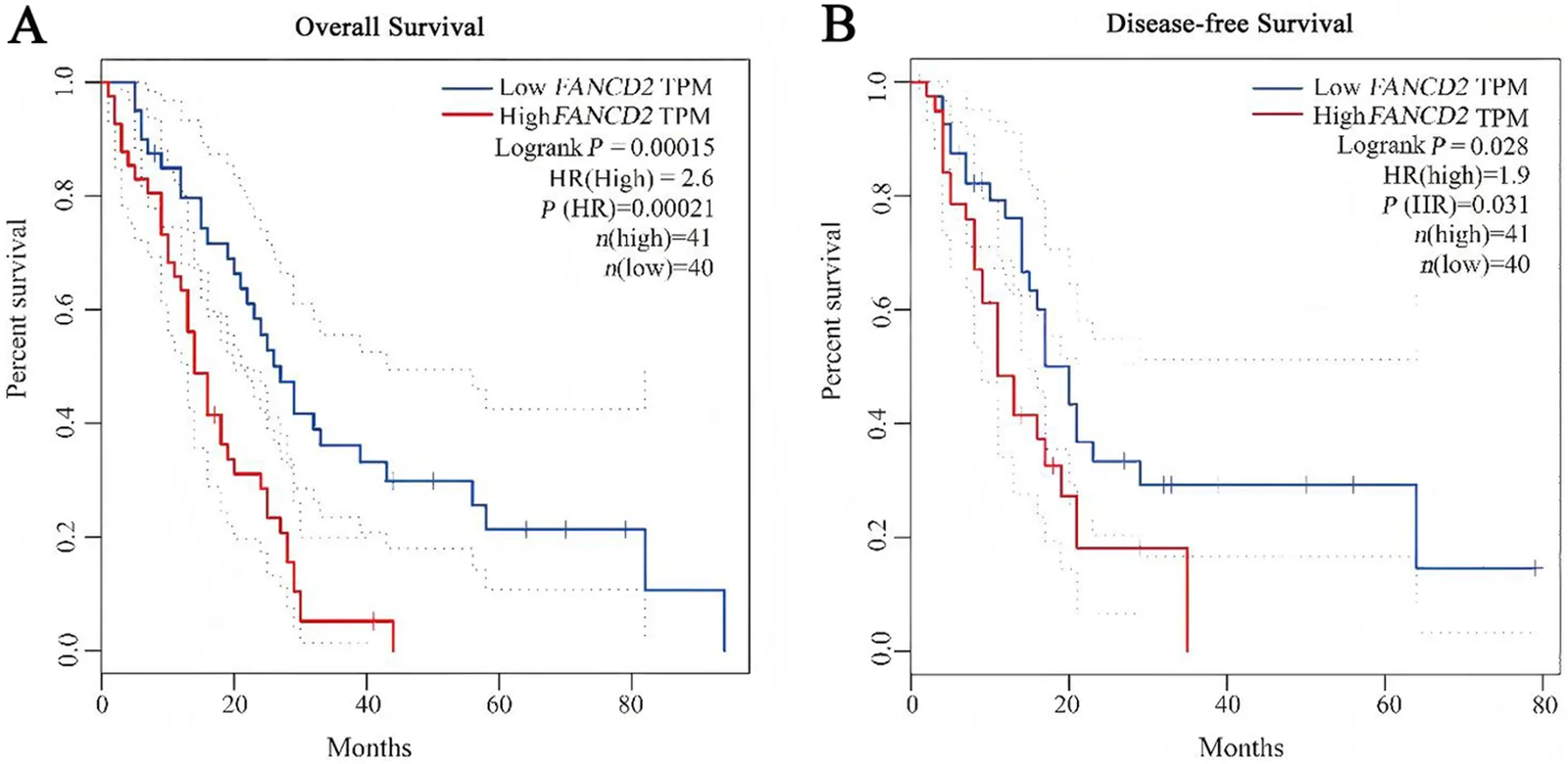
Correlation between FANCD2 expression and prognosis of PM patients. **(A)** Overall survival (OS). **(B)** Disease-free survival (DFS).

**TABLE 2:** Univariate and multivariable Cox proportional-hazards analyses for overall survival in patients with PM.

Clinicopathological parameters	Univariate analysis		Multivariate analysis	
	HR (95% CI)	P	HR (95% CI)	P
Age (≥60 vs. <60 years)	1.65 (0.96–2.81)	0.068	1.14 (0.64–2.02)	0.662
Sex (male vs. Female)	0.89 (0.49–1.59)	0.689	0.75 (0.40–1.42)	0.378
AJCC stage (vs. Stage I)	—	—	—	—
Stage II	0.63 (0.26–1.51)	0.301	0.83 (0.33–2.08)	0.691
Stage III	0.76 (0.36–1.59)	0.462	0.70 (0.31–1.55)	0.376
Stage IV	0.71 (0.30–1.67)	0.429	0.77 (0.30–1.97)	0.588
T stage (vs. T1)	—	—	—	—
T2	0.98 (0.47–2.03)	0.950	—	—
T3	1.06 (0.52–2.15)	0.880	—	—
T4	0.77 (0.33–1.84)	0.563	—	—
N stage (vs. N0)	—	—	—	—
N1	0.92 (0.44–1.91)	0.822	—	—
N2	0.81 (0.46–1.41)	0.446	—	—
N3	1.75 (0.54–5.73)	0.353	—	—
M stage (vs. M0)	—	—	—	—
M1 staging	1.86 (0.44–7.82)	0.399	—	—
Histological subtype (vs. Epithelioid)	—	—	—	—
Biphasic	**1.98 (1.18–3.33)**	**0.010**	**2.23 (1.26–3.97)**	**0.006**
Sarcomatoid	6.80 (0.89–52.25)	0.065	3.62 (0.42–31.52)	0.244
FANCD2 expression (high vs. Low)	**2.84 (1.73–4.66)**	**<0.001**	**3.10 (1.83–5.27)**	**<0.001**

Cox proportional hazards regression was performed using overall survival as the endpoint. Age was categorized as ≥60 versus <60 years, and FANCD2 expression was dichotomized at the median. Female sex, AJCC stage I, epithelioid histology, and low FANCD2 expression were used as the corresponding reference categories. The multivariable model included FANCD2 expression, age group, sex, AJCC stage, and histological subtype. T, N, and M categories were not entered separately into the multivariable model to avoid redundant adjustment with the composite AJCC stage. Because of the very small number of sarcomatoid cases, the corresponding hazard-ratio estimate should be interpreted with caution. HR, hazard ratio; CI, confidence interval; AJCC, American Joint Committee on Cancer. Bold values indicate statistically significant differences (P < 0.05).

### 3.4 *FANCD2* mRNA expression and immune infiltration in PM

Analysis of the TME revealed a higher stromal score in the high *FANCD2* expression group compared to the low-expression group, with no significant difference in immune scores (Figure 3A). Furthermore, *FANCD2* expression exhibited distinct correlations with immune cell infiltration (Figure 3B). Specifically, it was positively correlated with the infiltration of M0, M1, and M2 macrophages. Conversely, it was inversely correlated with the infiltration of activated NK cells, activated dendritic cells, and plasma cells. Notably, in mesothelioma, *FANCD2* expression demonstrated a significant positive correlation with M2 macrophage infiltration (Figure 3C) and significant negative correlations with both activated NK cell (Figure 3D) and activated dendritic cell infiltration (Figure 3E). Additionally, we screened the PM expression database to identify genes correlated with *FANCD2* (Figure 4A). Subsequent co-expression analysis revealed significant positive correlations between *FANCD2* and several genes, including cancerous inhibitor of protein phosphatase 2A (*CIP2A*), timeless circadian regulator gene (*TIMELESS*), DNA polymerase θ (*POLQ*), *FANCI*, establishment of sister chromatid cohesion N-acetyltransferase 2 (*ESCO2*), and *BUB1* mitotic checkpoint serine/threonine kinase B (*BUB1B*) (Figure 4B). Conversely, significant negative correlations were observed with genes such as interferon-induced transmembrane protein 2 (*IFITM2*), tumor suppressor candidate 1 (*TUSC1*), transcription elongation factor A like 2 (*TCEAL2*), phospholipase A2 group IB (*PLA2G1B*), and cytochrome b5 type A (*CYB5A*) (Figure 4A). Furthermore, analysis of immune checkpoint genes demonstrated that *FANCD2* expression was positively correlated with the levels of CD276, TNF receptor superfamily member 25 (*TNFRSF25*), and neuropilin 1 (*NRP1)* (Figures 4C,D).

**FIGURE 3:**
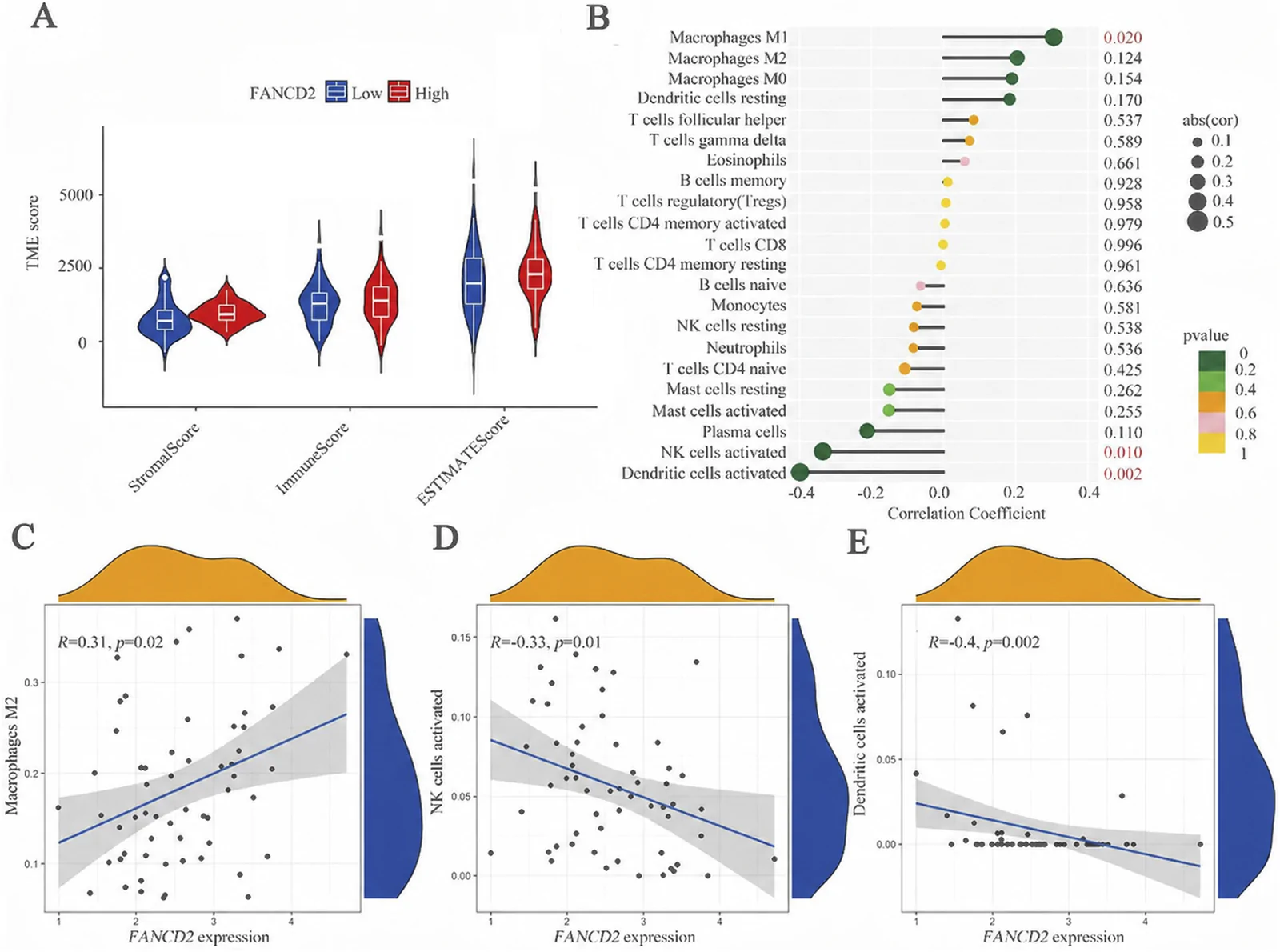
Association between FANCD2 expression and computationally estimated immune-cell proportions in PM. **(A)** Association of FANCD2 expression with tumor microenvironment (TME) scores. **(B)** Associations between FANCD2 expression and the estimated proportions of different immune-cell populations. **(C–E)** Scatter plots showing the correlations of FANCD2 expression with the estimated proportions of M2 macrophages **(C)**, activated NK cells **(D)**, and activated dendritic cells **(E)**. In panels C–E, the orange and blue marginal density curves represent the distributions of FANCD2 expression and the corresponding estimated immune-cell proportions, respectively. The marginal density curves are provided to visualize the univariate distribution of each variable and do not represent additional statistical tests. Correlations were assessed using Spearman correlation analysis. Multiple testing across immune-cell types was controlled using the Benjamini–Hochberg method. *P < 0.05, **P < 0.01, ***P < 0.001. The orange and blue marginal density curves show the distributions of *FANCD2* expression and the corresponding immune-cell infiltration estimates, respectively.

**FIGURE 4:**
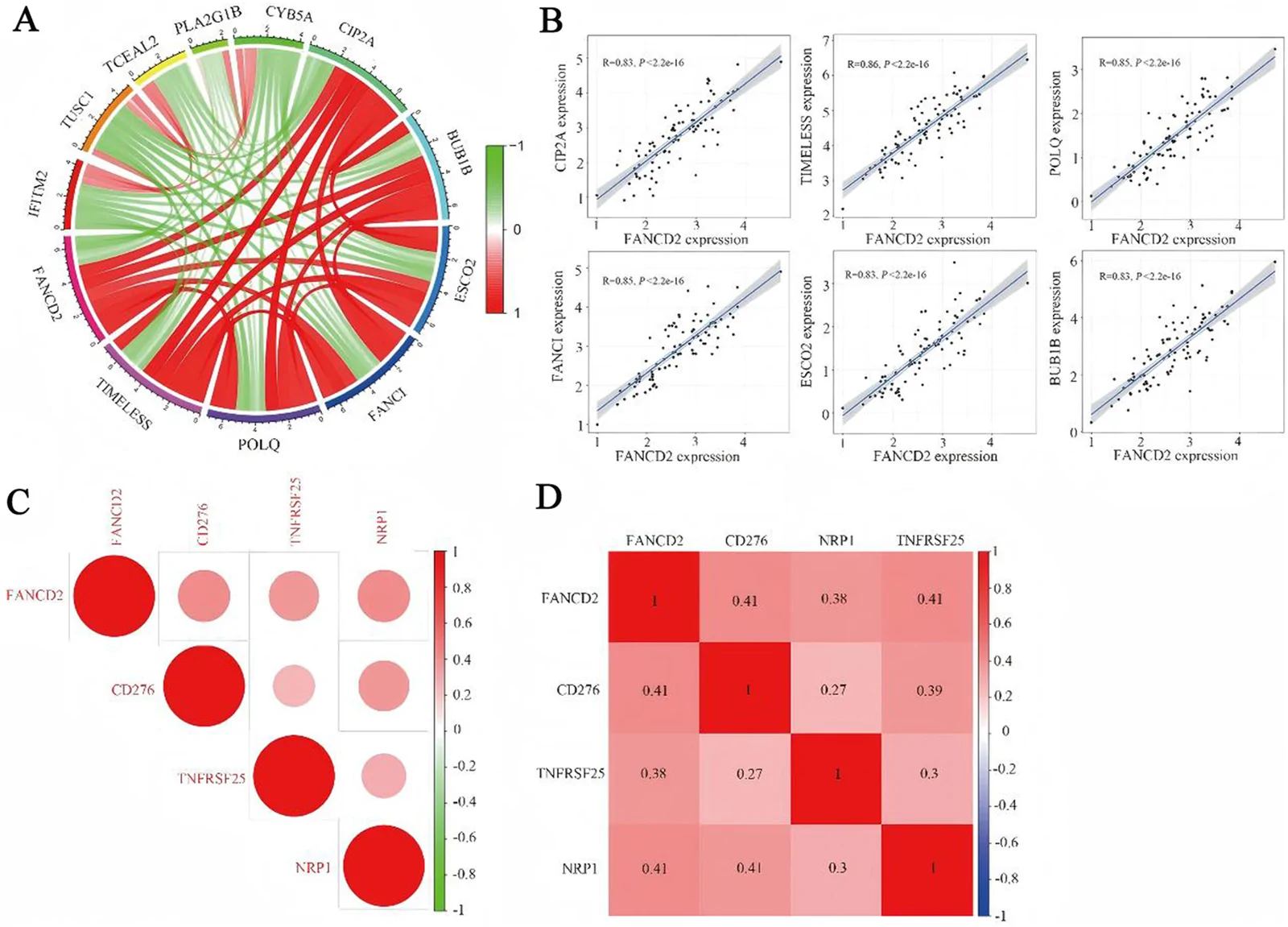
Association of *FANCD2* expression with co-expressed genes and immune checkpoint-related molecules in PM. **(A)** Correlation network showing genes positively or negatively associated with *FANCD2* expression in the TCGA-MESO cohort. **(B)** Scatter plots showing representative genes positively correlated with *FANCD2*, including *CIP2A*, *TIMELESS*, *POLQ*, *FANCI*, *ESCO2*, and *BUB1B*. **(C)** Bubble plot showing the correlations between *FANCD2* and immune checkpoint-related genes. **(D)** Correlation heatmap showing positive associations among *FANCD2*, *CD276*, *TNFRSF25*, and *NRP1* expression levels. Red indicates positive correlation, whereas green indicates negative correlation. Correlations were analyzed using Pearson correlation analysis. *P < 0.05 was considered statistically significant.

### 3.5 Enrichment analysis

GO enrichment analysis showed that FANCD2-associated genes were significantly enriched in cellular components including the spindle, chromosome/centromeric regions, and intermediate filaments. FANCD2-associated genes were enriched for molecular functions including microtubule binding, serine/threonine kinase activity, and motor activity (Figure 5A). KEGG enrichment analysis indicated that FANCD2-associated genes were enriched in pathways including the cell cycle and oocyte meiosis (Figure 5B). Subsequent GSEA of PM expression data demonstrated that the high *FANCD2* expression group was enriched in pathways related to adhesion junctions, focal adhesion, and cardiomyopathy (Figure 5C). Conversely, the low-expression group was primarily associated with pathways involving complement activation, humoral immune responses, and immunoglobulin-related processes (Figure 5D).

**FIGURE 5:**
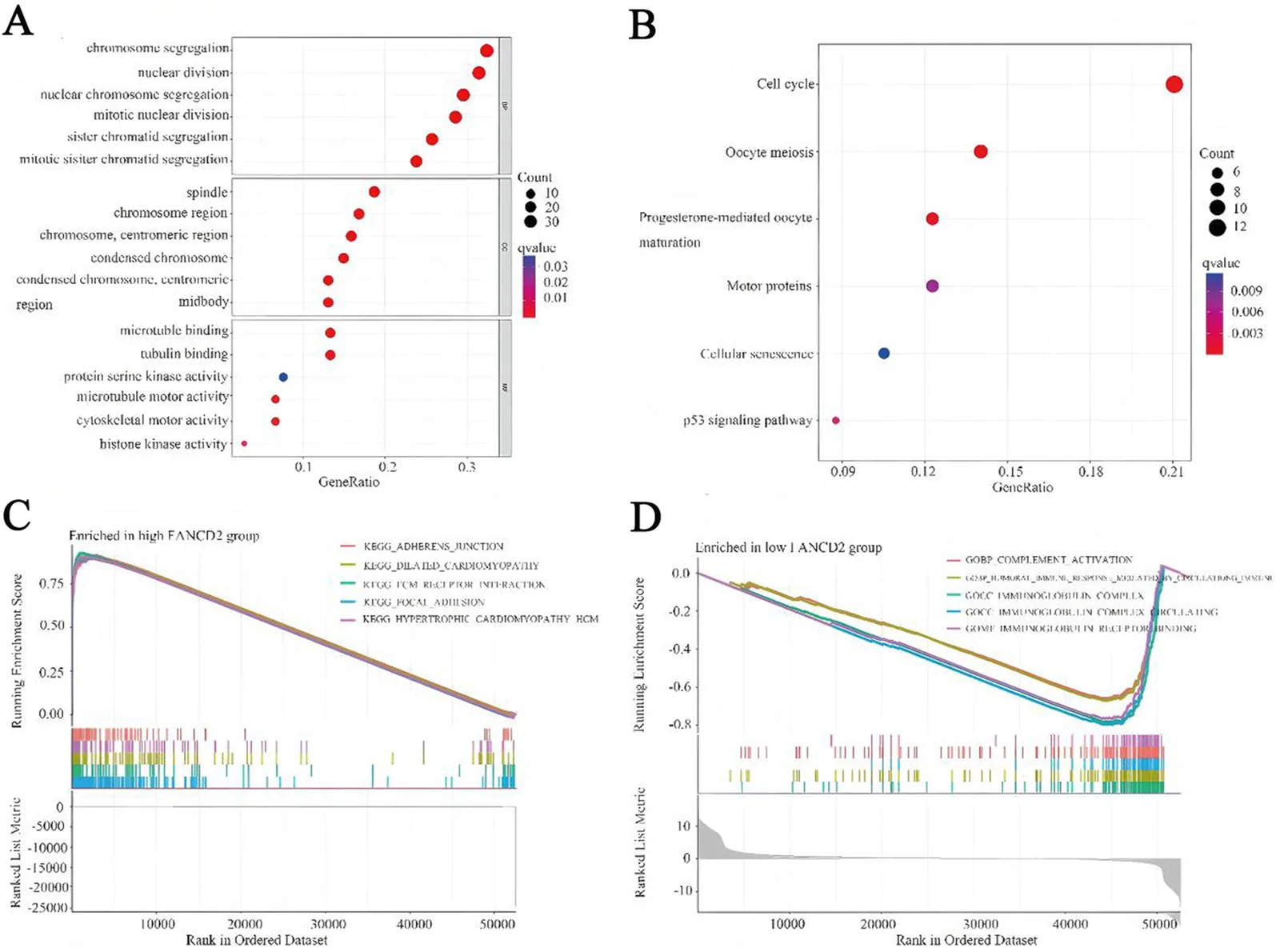
Functional enrichment analysis of *FANCD2* expression. **(A)** GO enrichment analysis, including biological process (BP), molecular function (MF), and cellular components (CC) analysis. **(B)** KEGG enrichment analysis. **(C)** GSEA showing pathways enriched in the high-FANCD2 expression group. **(D)** GSEA showing pathways enriched in the low-FANCD2-expression group.

### 3.6 FANCD2 mRNA and protein levels are elevated in PM tissues and cell lines

A marked upregulation of *FANCD2* mRNA was observed in ten PM tissues compared to paired pleural mesothelial tissues (*P* < 0.001, Figure 6A). Western blot analysis also demonstrated a marked upregulation of FANCD2 protein in PM tissues compared to paired adjacent non-tumor pleural mesothelial tissues (*P* < 0.001, Figures 6B,C). qRT-PCR results further confirmed that, compared to MeT-5A, *FANCD2* mRNA expression was significantly upregulated in four different PM cell lines (*P* < 0.01; *P* < 0.001, Figure 6D), with NCI-H2052 cells showing the highest mRNA expression level. Similarly, Western blot analysis also showed that, compared to MeT-5A, FANCD2 protein expression was significantly elevated in the four different PM cell lines (*P* < 0.01; *P* < 0.001, Figures 6E,F), and the highest protein expression level was also observed in NCI-H2052 cells.

**FIGURE 6:**
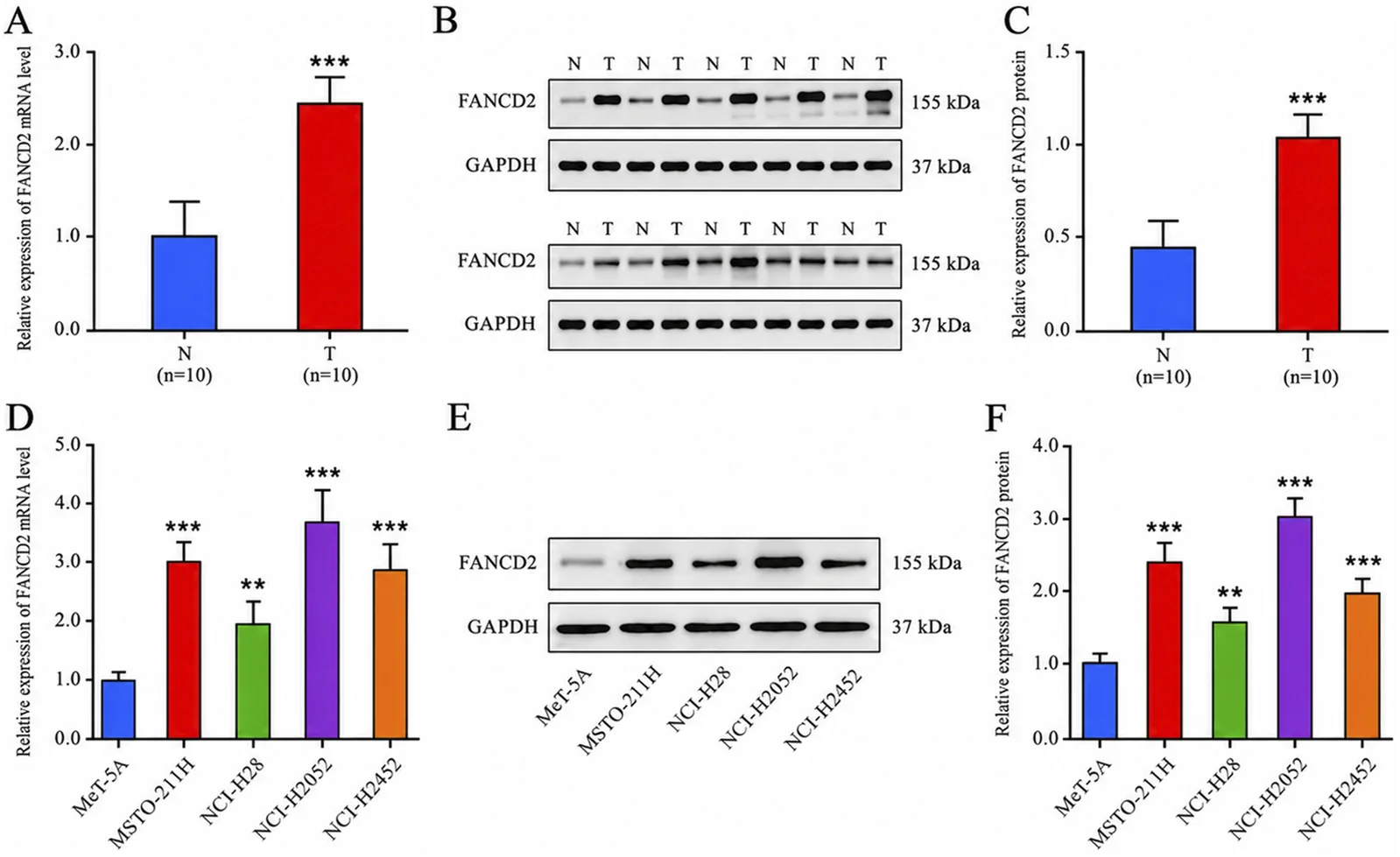
The expression of *FANCD2* mRNA and protein in the PM tissues and PM cells. **(A)** qRT-PCR detection of *FANCD2* mRNA expression in PM tissues and paired adjacent non-tumor pleural mesothelial tissues. **(B,C)** Western blotting detection of FANCD2 protein expression in PM tissues and paired adjacent non-tumor pleural mesothelial tissues. **(D)** qRT-PCR detection of *FANCD2* mRNA expression in the normal pleural mesothelial cell line MeT-5A and four PM cell lines. **(E–F)** Western blotting detection of FANCD2 protein expression in the normal pleural mesothelial cell line MeT-5A and four PM cell lines. **P < 0.01, ***P < 0.001.

### 3.7 Knockdown of *FANCD2* gene significantly inhibits NCI-H2052 cell proliferation and reduces wound-closure capacity

Because NCI-H2052 cells exhibited the highest FANCD2 expression among the examined PM cell lines, they were selected for subsequent functional experiments. Following transient siRNA-mediated FANCD2 knockdown in NCI-H2052 cells, FANCD2 mRNA and protein levels were significantly reduced (*P* < 0.01, Figures 7A,B). CCK-8 absorbance increased over time in all groups; however, FANCD2 knockdown significantly reduced cell viability relative to the si-NC group at 48 and 96 h *(P* < 0.01; Figure 7C). Consistently, colony formation was significantly reduced in both the si-FANCD2-1 and si-FANCD2-2 groups (*P* < 0.01; Figures 7D,E). FANCD2 knockdown also significantly reduced wound closure at 48 h compared with the si-NC group (P < 0.01; Figures 7F,G). Because a proliferation inhibitor was not used during the wound-healing assay, the reduced wound closure may reflect combined effects on cell proliferation, survival, and motility rather than a migration-specific effect.

**FIGURE 7:**
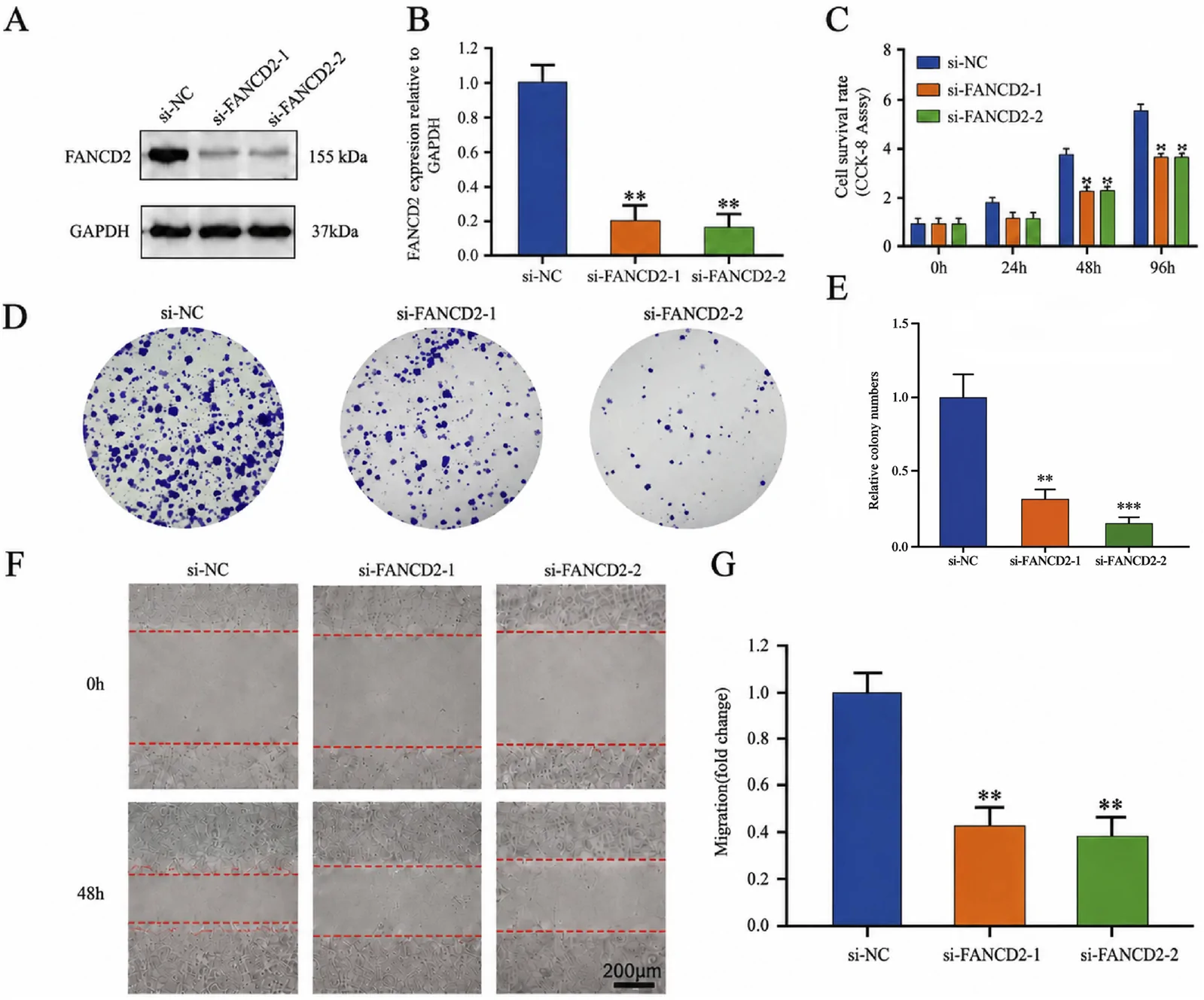
Effects of transient FANCD2 knockdown on cell viability, colony formation, and wound-closure capacity in NCI-H2052 cells. **(A)** qRT-PCR analysis of FANCD2 mRNA expression. **(B)** Western blotting analysis of FANCD2 protein expression. **(C)** Cell viability measured using the CCK-8 assay. **(D,E)** Representative images and quantitative analysis of colony formation. **(F,G)** Representative images and quantitative analysis of wound closure at 48 h. Data are presented as the mean ± SD from three independent biological experiments (n = 3). CCK-8 data were analyzed using two-way ANOVA followed by Dunnett’s multiple-comparisons test. Other comparisons were analyzed using one-way ANOVA followed by Dunnett’s multiple-comparisons test. **P < 0.01 and ***P < 0.001 versus the si-NC group.

### 3.8 Effects of *FANCD2* knockdown on cell cycle distribution in NCI-H2052 cells

FANCD2 knockdown altered the cell-cycle distribution and induced a significant G1/S phase cell cycle arrest of NCI-H2052 cells, as indicated by an increased proportion of cells in the G0/G1 phase and a corresponding decrease in the S phase (*P* < 0.001; Figures 8A,B). Western blotting showed significantly decreased expression of CDK6, CDK4, and Cyclin D1 following FANCD2 knockdown (*P* < 0.01 or *P* < 0.001; Figures 8C,D). These findings indicate that the reduced proliferative phenotype following FANCD2 depletion was accompanied by G0/G1-phase arrest with impaired G1-to-S transition and decreased expression of selected cell-cycle-related proteins.

**FIGURE 8:**
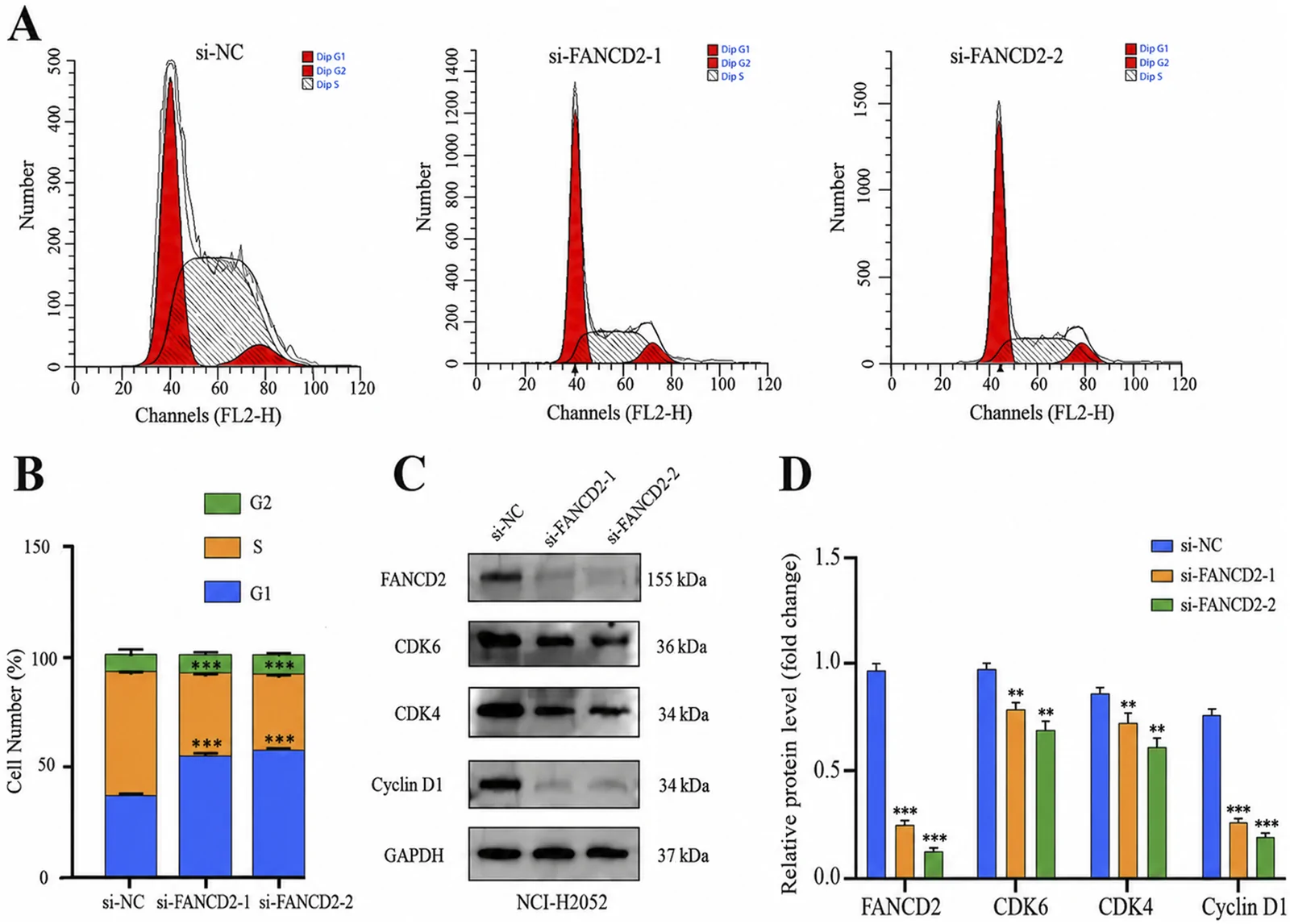
Effects of transient FANCD2 knockdown on cell-cycle distribution in NCI-H2052 cells. **(A)** Representative flow-cytometric plots of cell-cycle distribution. **(B)** Quantitative analysis of the proportions of cells in the G0/G1, S, and G2/M phases. **(C,D)** Representative Western blots and quantitative analysis of CDK6, CDK4, and Cyclin D1 expression. Data are presented as the mean ± SD from three independent biological experiments (n = 3). The Western blot images shown are representative of three independent biological experiments; quantitative values were calculated from all three experiments after normalization to the corresponding loading control.Statistical comparisons were performed using one-way ANOVA followed by Dunnett’s multiple-comparisons test. *P < 0.05, **P < 0.01, and ***P < 0.001 versus the si-NC group.

### 3.9 Effects of *FANCD2* knockdown on apoptosis in NCI-H2052 cells

Flow-cytometric analysis using Annexin V-FITC/PI staining showed that transient FANCD2 knockdown markedly increased apoptosis in NCI-H2052 cells (Figure 9A). The total apoptotic proportion, calculated as the sum of early and late apoptotic cells, was approximately 4.2% in the si-NC group, compared with 19.5% in the si-FANCD2-1 group and 24.5% in the si-FANCD2-2 group. Consistently, quantitative analysis confirmed that both FANCD2-targeting siRNAs significantly increased the apoptotic proportion compared with si-NC (both **P < 0.001), with si-FANCD2-2 producing the greatest increase (Figure 9B). Western blotting further showed increased levels of cleaved caspase-3, cleaved caspase-8, cleaved caspase-9, and cleaved PARP after FANCD2 knockdown (*P* < 0.01 or *P* < 0.001; Figures 9C,D). These findings indicate that the increased apoptosis following FANCD2 depletion was accompanied by activation of caspase/PARP-related apoptotic markers. However, these results do not establish direct regulation of these proteins by FANCD2 or demonstrate the independent activation of specific death-receptor or mitochondrial apoptotic pathways.

**FIGURE 9:**
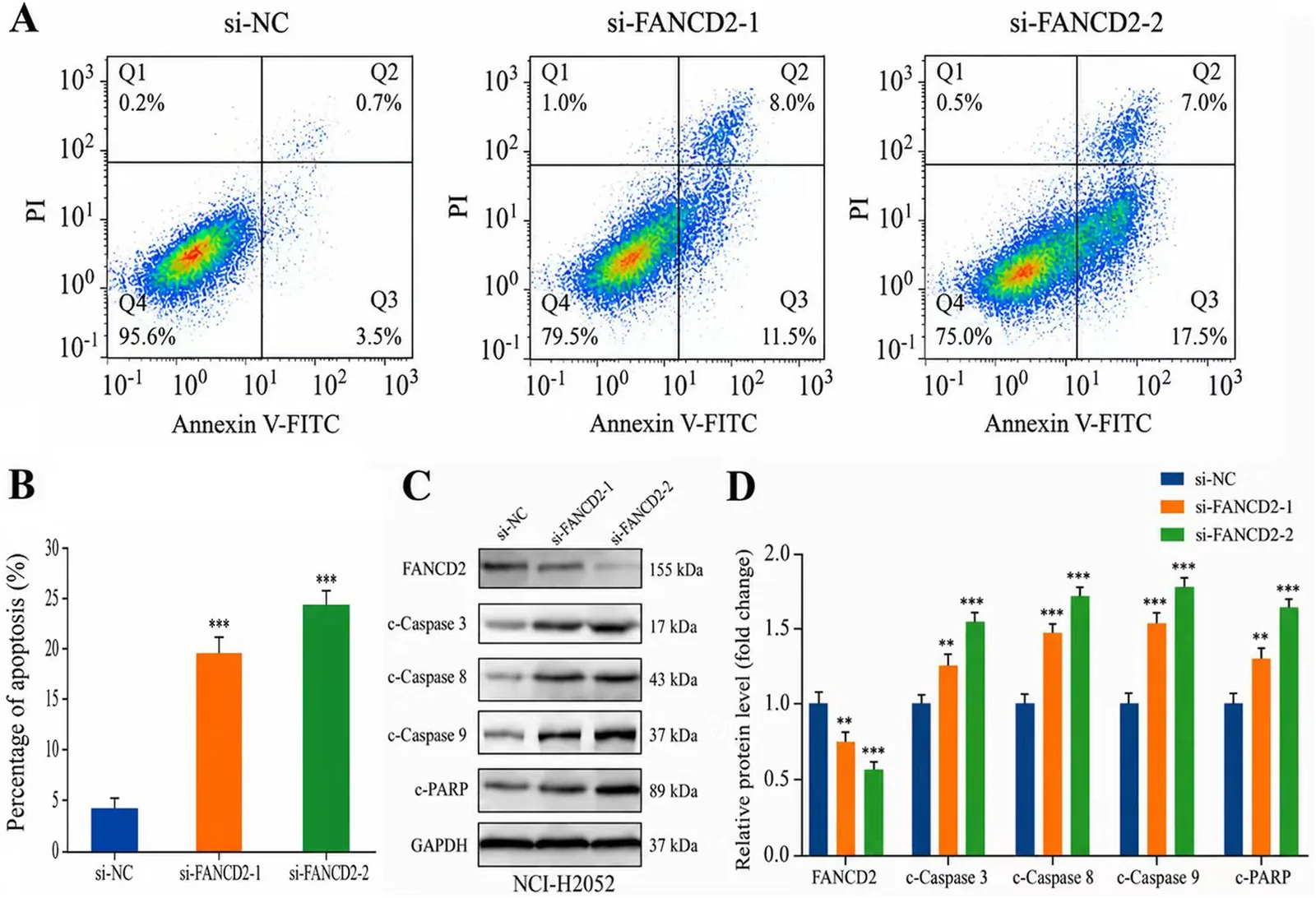
Effects of transient FANCD2 knockdown on apoptosis in NCI-H2052 cells. **(A)** Representative flow-cytometric plots of Annexin V-FITC/PI staining. **(B)** Quantitative analysis of the apoptotic cell proportion. **(C,D)** Representative Western blots and quantitative analysis of cleaved caspase-3, cleaved caspase-8, cleaved caspase-9, and cleaved PARP. Data are presented as the mean ± SD from three independent biological experiments (n = 3). Statistical comparisons were performed using one-way ANOVA followed by Dunnett’s multiple-comparisons test. **P < 0.01 and ***P < 0.001 versus the si-NC group.

### 3.10 FANCD2 restoration and caspase inhibition partially reverse the effects of FANCD2 knockdown

To further determine whether the effects of FANCD2 depletion involved caspase-dependent apoptotic signaling, FANCD2 rescue and Z-VAD-FMK inhibition experiments were performed in NCI-H2052 cells. FANCD2 knockdown markedly suppressed cell viability over time, whereas restoration of FANCD2 partially rescued this reduction. Z-VAD-FMK treatment also partially restored the viability of FANCD2-silenced cells, while treatment with Z-VAD-FMK alone had little effect on control cells (Figure 10A). Consistent with the CCK-8 results, FANCD2 knockdown substantially reduced colony-forming capacity. FANCD2 overexpression partially restored colony formation, and inhibition of caspase activity by Z-VAD-FMK also partially reversed the suppressive effect of FANCD2 knockdown (Figures 10B,C). Annexin V-FITC/PI flow cytometry further showed that FANCD2 knockdown markedly increased the proportion of apoptotic NCI-H2052 cells. Restoration of FANCD2 significantly reduced FANCD2 knockdown-induced apoptosis. Similarly, Z-VAD-FMK treatment decreased the apoptotic proportion of FANCD2-silenced cells, whereas Z-VAD-FMK alone produced only a minor change in control cells (Figures 10D,E). Western blot analysis confirmed efficient FANCD2 depletion and restoration. FANCD2 knockdown was accompanied by increased levels of cleaved caspase-3, cleaved caspase-8, cleaved caspase-9, and cleaved PARP. These changes were partially reversed by restoration of FANCD2 expression. Z-VAD-FMK treatment also attenuated the increases in apoptosis-associated proteins induced by FANCD2 knockdown without restoring FANCD2 expression itself (Figures 10F,G). Collectively, these findings demonstrate that restoration of FANCD2 partially reverses the growth inhibition and apoptosis induced by FANCD2 depletion. Furthermore, pharmacological caspase inhibition partially rescues these phenotypes, supporting the involvement of caspase-dependent apoptotic signaling in the cellular effects of FANCD2 knockdown.

**FIGURE 10:**
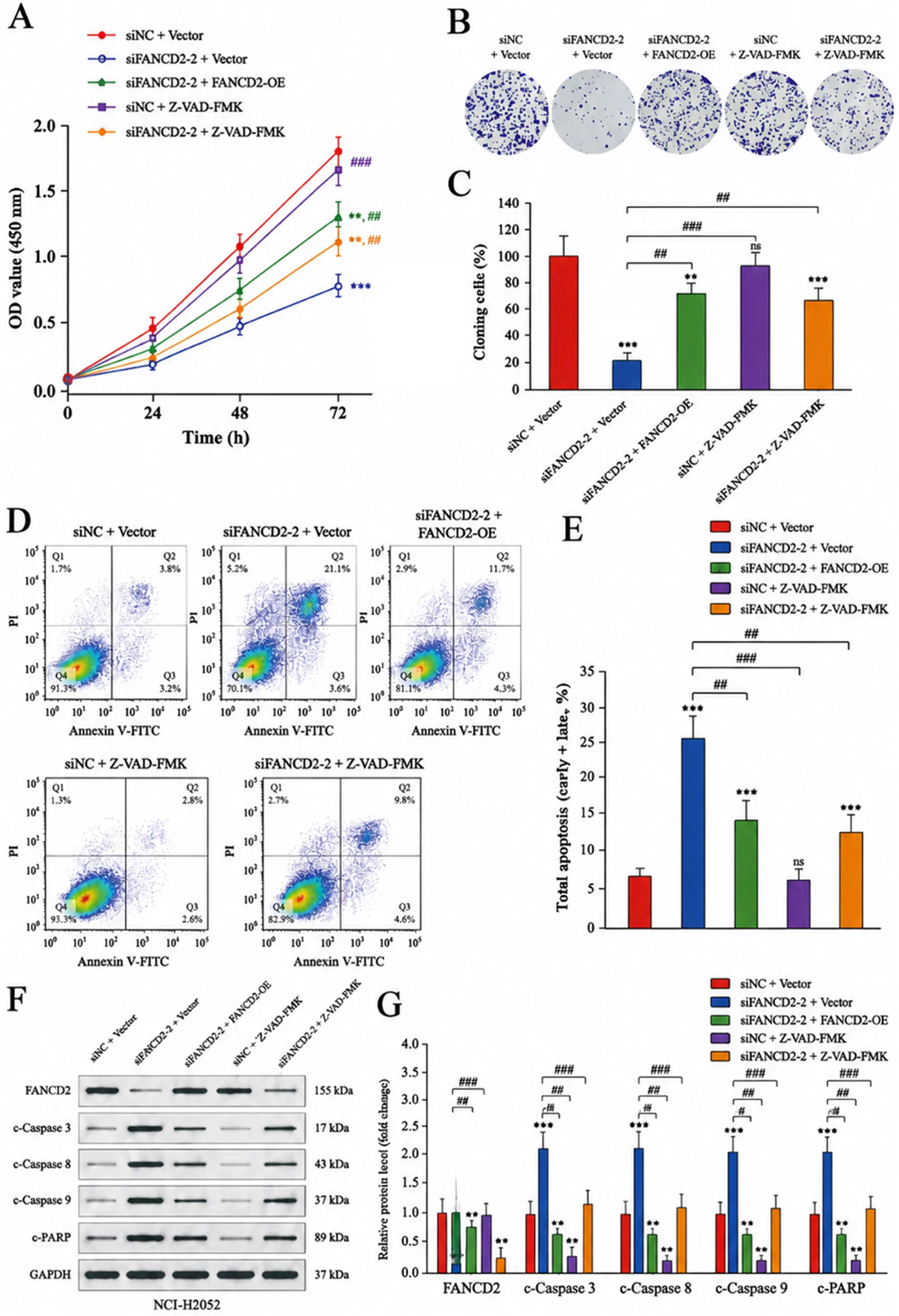
FANCD2 restoration and caspase inhibition partially reverse the effects of FANCD2 knockdown in NCI-H2052 cells. **(A)** CCK-8 assay showing cell viability after the indicated treatments. **(B,C)** Representative colony formation images and quantitative analysis. **(D,E)** Representative Annexin V-FITC/PI flow cytometry plots and quantification of total apoptotic cells. **(F,G)** Western blot analysis and densitometric quantification of FANCD2, cleaved caspase-3, cleaved caspase-8, cleaved caspase-9, and cleaved PARP; GAPDH served as the loading control. FANCD2 knockdown suppressed cell growth and increased apoptosis, whereas FANCD2 overexpression or Z-VAD-FMK treatment partially reversed these effects. *P < 0.05, **P < 0.01, ***P < 0.001 versus siNC + Vector; ^#^P < 0.05, ^##^P < 0.01, ^###^P < 0.001 versus siFANCD2 + Vector.

## 4 Discussion

Most patients with PM present with locally advanced disease, necessitating an integrated, multimodal approach to treatment. This therapeutic strategy is determined by the disease stage, histology, and the patient’s overall performance status (Brims et al., 2019). Given the constraints of current metastatic treatments and the significant spatiotemporal genetic diversity in solid tumors, therapeutic efficacy is often low and relapse rates high, which result in an overall poor prognosis. Hence, there remains a need to identify and validate clinically useful biomarkers for the diagnosis, prognosis, and treatment stratification of PM (Grant et al., 2025). Studies have shown that *FANCD2* is involved in regulating tumorigenesis, apoptosis, and other processes in cancer (Xia et al., 2015), such as glioblastoma (Song et al., 2022), esophageal squamous cell carcinoma (ESCC) (Lei et al., 2020) and LUAD (Miao et al., 2022). As a gene associated with ferroptosis, *FANCD2* can regulate the ferroptosis process. Ferroptosis is closely related to the progression of various cancer types and plays a key role in PM, with *FANCD2* being identified as an important regulatory factor in this process and significantly impacting disease prognosis (Wang et al., 2024). *FANCD2* has been implicated in PM progression, where its high expression not only correlates with unfavorable patient outcomes but also, when knocked down, sensitizes cancer cells to ferroptosis inducers (Wang et al., 2024).

By integrating clinical and transcriptomic data from PM patients in the TCGA and GEO cohorts, we comprehensively evaluated the expression profile of the *FANCD2* gene. Integrated bioinformatics analysis revealed that *FANCD2* was significantly elevated in PM tissues and its expression correlated with key clinical parameters, including tumor T-stage. The demographic and epidemiological characteristics of PM have been reported previously (Brims, 2021)**.** Univariate Cox analysis identified high *FANCD2* expression levels, alongside the pathological type, as factors significantly associated with adverse prognosis in PM. Multivariate Cox regression showed that high *FANCD2* expression remained associated with poor prognosis after adjustment for available clinicopathological variables. However, because several important clinical and molecular factors were unavailable or incompletely documented in the TCGA-MESO dataset, residual confounding cannot be excluded. Therefore, *FANCD2* should be considered an exploratory candidate prognostic indicator that requires further validation in larger cohorts with comprehensive clinical and genomic annotations. Separately, tumor-infiltrating immune cells are established as fundamental regulators of tumor progression and the efficacy of immune checkpoint inhibition (Petitprez et al., 2020). Higher FANCD2 expression was associated with a greater computationally estimated proportion of M2 macrophages. Because M2-like macrophages have been associated with immunosuppressive tumor microenvironments (TME), this observation raises a hypothesis regarding a possible relationship between FANCD2 expression and the immune contexture of PM (Song et al., 2024); however, no causal relationship can be inferred from the present analysis. Successful immunotherapy is determined by adequate infiltration of immune cells into the TME and the expression of critical immune checkpoint molecules (Li et al., 2024). Analysis of immune checkpoints showed that *FANCD2* expression was strongly correlated with several molecules, including *CD276*, *TNFRSF25*, and *NRP1*, among which *CD276* promotes immune evasion and assists cancer cells in escaping surveillance through its high expression (Flem-Karlsen et al., 2018). *NRP1* is a key regulator of immune suppression in TME, where it stabilizes Tregs and inhibits CD8^+^ T cell function, thereby positioning it as an important target for cancer immunotherapy (Chuckran et al., 2021; Liu et al., 2020). Because the immune-cell estimates were inferred computationally from bulk transcriptomic data, the observed associations do not demonstrate that *FANCD2* directly recruits or regulates M2 macrophages, NK cells, or dendritic cells. Residual confounding by tumor purity, stromal content, histological composition, and other molecular features cannot be excluded. These immune associations should therefore be regarded as hypothesis-generating and require validation using spatial, single-cell, or tissue-based methods. We next performed GSEA on the differentially expressed genes and found that *FANCD2* may influence the biological functions of PM through pathways such as cell cycle, cellular senescence, and oocyte meiosis. Previous studies have shown that knockdown of *FANCD2* expression could increase DNA damage and reduce the number of S-phase cells, thereby decreasing tumor cell proliferation, which is consistent with the gene enrichment results for *FANCD2* in cell cycle, oocyte meiosis, and DNA replication pathways in PM (Zheng et al., 2022). In addition, several genes, including *CIP2A*, *TIMELESS*, *POLQ*, *FANCI*, *ESCO2*, and *BUB1B*, were found to be significantly positively correlated with *FANCD2* expression. Among them, CIP2A, an endogenous inhibitor of protein phosphatase 2A (PP2A), promotes oncogenesis by inhibiting PP2A-mediated dephosphorylation of protein kinase B and stabilizing the MYC oncoprotein. Overexpression of *CIP2A* is common in PM patients. This finding provides important theoretical support for improving PM patient prognosis through personalized combination therapies (Pippa et al., 2020). Related research also suggests that *ESCO2* is highly expressed in several malignant tumors, such as LIHC, PAAD, LUAD, and MESO, and its overexpression correlates with significantly shorter patient survival, making it a reliable prognostic predictor. Furthermore, ESCO2 is involved in the regulatory network of the cell cycle and tumor cell proliferation (Huang et al., 2024). *FANCD2* functions as an oncogenic driver in LUAD, enhancing tumor cell proliferation by regulating the cell cycle, while also promoting invasion and migration. The growing body of evidence from various human cancers highlights the potential of *FANCD2* to serve as both a biomarker and a therapeutic target (Xie et al., 2024). The enrichment of cell-cycle, chromosome-segregation, and mitotic processes suggests that high *FANCD2* expression may partly represent a highly proliferative tumor phenotype. Therefore, *FANCD2* should not be interpreted solely as an independent oncogenic driver on the basis of the current association analyses. Future studies should determine whether its prognostic association persists after adjustment for *MKI67*, *PCNA*, *TOP2A*, or a composite proliferation signature.

To further explore whether *FANCD2* regulates the proliferation of NCI-H2052 cells through the cell cycle, *FANCD2* was transiently silenced in NCI-H2052 cells using two independent siRNAs. The experimental results showed that the proliferation and wound-closure capacity of NCI-H2052 cells with knockdown of *FANCD2* gene expression were significantly reduced. These findings indicate that FANCD2 depletion impairs the proliferative and wound-closure capacities of NCI-H2052 cells, supporting a functional contribution of FANCD2 to these cellular phenotypes. *FANCD2* knockdown induced a G0/G1-phase arrest with impaired G1-to-S transition in NCI-H2052 cells, as indicated by a markedly increased proportion of cells in the G0/G1 phase and a concurrent reduction in S phase cells, which was analyzed via flow cytometry. Furthermore, *FANCD2* knockdown significantly downregulated key G1/S transition regulators, including CDK4, CDK6, and cyclin D1. These changes were accompanied by G0/G1-phase accumulation and impaired G1-to-S transition, suggesting that altered cell-cycle regulation may contribute to the growth suppression observed after FANCD2 depletion in NCI-H2052 cells. *FANCD2* may represent a candidate molecule for further investigation in therapeutic development (Røe et al., 2010). In the early stages of cancer, genomic instability increases, and FANCD2 may be activated due to increased DNA damage (e.g., through ubiquitination modification). Radiotherapy and certain chemotherapeutic agents (such as cisplatin and mitomycin) kill tumor cells by inducing DNA damage, while the *FANCD2*-mediated repair pathway reduces the tumor’s sensitivity to these treatments. If *FANCD2* is highly expressed in tumors, it may suggest strong DNA repair capacity and resistance to drugs like cisplatin, indicating the need to adjust treatment regimens. Further study has shown that *FANCD2* expression is closely related to proliferative markers such as Ki-67, Cyclin A, and mitotic activity index (MAI). FANCD2 and Ki-67 are co-expressed in invasive breast cancer cells (van der Groep et al., 2008), suggesting that combined detection of FANCD2 with other markers (e.g., p53, Ki-67, DNA damage-related genes) could improve the accuracy of prognosis assessment. The biological significance of FANCD2 in PM should also be considered within the broader genomic and DNA-damage-response landscape of this malignancy. PM is characterized by recurrent alterations in tumor-suppressor and genome-maintenance genes, including BAP1, CDKN2A, NF2, TP53, and ATM. Together with chronic asbestos-associated oxidative stress, DNA damage, and replication stress, these alterations may create a cellular environment in which efficient DNA-damage tolerance and repair become particularly important for tumor-cell survival. FANCD2 is a central effector of the Fanconi anemia pathway and contributes to the repair of DNA interstrand crosslinks, stabilization of stalled replication forks, and maintenance of genomic integrity. Accordingly, elevated FANCD2 expression in PM may reflect, at least in part, an adaptive response that enables tumor cells to tolerate persistent genotoxic and replication stress. This interpretation is consistent with the growth suppression and cell-cycle alterations observed following FANCD2 depletion in the present study, although we did not directly measure DNA-damage accumulation or replication-fork stability. Therefore, the present findings should not be interpreted as demonstrating a specific DNA-repair mechanism downstream of FANCD2 in PM. Further studies incorporating markers of DNA damage and replication stress, together with genetically defined PM models, will be required to clarify this possibility. Recently, a study has revealed that BAP1 and ubiquitin-specific protease 1 (USP1) cooperate to regulate FANCD2 stability and cell proliferation in mesothelioma cells. In contrast, depletion of *FANCD2* suppressed cell proliferation irrespective of BAP1 status, underscoring the essential role of FANCD2 in mesothelioma cell survival. These findings support a working model in which USP1-dependent maintenance of FANCD2 function becomes critical in the absence of functional *BAP1* (Suzuki et al., 2026). Our findings are consistent with the broader observation by Suzuki et al. (2026) that FANCD2 depletion can suppress mesothelioma-cell proliferation independently of BAP1 status. NCI-H2052 has been reported as a *BAP1*-wild-type mesothelioma cell line; however, the *BAP1* status of the specific cell batch used in this study was not independently verified. Therefore, our findings represent a FANCD2-associated phenotype in the NCI-H2052 cellular context and do not establish BAP1-dependent FANCD2 regulation. Further investigation using PM models with defined *BAP1* status will be important to clarify the relationship between BAP1 background and FANCD2 function.

Cleaved-caspase 3, cleaved-caspase 8, cleaved-caspase 9, and cleaved PARP are important markers of cell apoptosis (Zhang et al., 2020; Alasmari, 2024; Yang et al., 2025). In the death receptor pathway, the binding of death receptors to death ligands can form a death-inducing signaling complex (DISC), which promotes the autocatalytic cleavage of procaspase-8 to form active caspase-8, activates downstream caspase-3 and PARP, and subsequently triggers apoptosis (Moriwaki et al., 2021; Yuan et al., 2018). In the mitochondrial pathway, when cells are stimulated by apoptotic signals, cytochrome c released from mitochondria binds to apoptotic protease-activating factor 1 (Apaf-1), promoting apoptosome formation and subsequent activation of caspase-9. This initiates a cascade reaction, leading to caspase-3 activation and subsequent PARP cleavage, and leading to apoptosis (Hao et al., 2023; Hao et al., 2021). In the present study, FANCD2 knockdown increased the levels of cleaved caspase-8, cleaved caspase-9, cleaved caspase-3, and cleaved PARP, indicating enhanced caspase-associated apoptotic signaling. However, the concurrent increase in caspase-8 and caspase-9 does not demonstrate that the death-receptor and mitochondrial apoptotic pathways were independently or simultaneously activated. Crosstalk between apoptotic signaling pathways may also contribute to activation of these caspases. Because upstream pathway-specific events were not directly examined, the present data do not allow us to distinguish the relative contribution of the extrinsic and intrinsic apoptotic pathways. Our rescue experiments further support the involvement of caspase-dependent apoptotic signaling in the biological effects of FANCD2 depletion. Restoration of FANCD2 partially reversed the reductions in cell viability and colony formation and attenuated the increase in apoptosis induced by FANCD2 knockdown. Similarly, treatment with the pan-caspase inhibitor Z-VAD-FMK partially rescued these phenotypic changes and reduced the activation of apoptosis-related markers, including cleaved caspase-3, cleaved caspase-8, cleaved caspase-9, and cleaved PARP. These findings suggest that caspase-dependent apoptosis contributes, at least in part, to the growth-suppressive effects of FANCD2 depletion in NCI-H2052 cells. However, these results do not establish a direct regulatory interaction between FANCD2 and the caspase/PARP pathway, and the precise molecular mechanisms linking FANCD2 loss to apoptotic signaling require further investigation.

The enrichment of cell-cycle, chromosome-segregation, and mitotic processes suggests that high *FANCD2* expression may partly represent a highly proliferative tumor phenotype. However, the regulatory effect of FANCD2 on PM cell proliferation, wound-closure capacity, and apoptosis may be related to multiple signaling pathways and gene interactions. This study has not yet elucidated its exact molecular mechanism, and further exploration of its regulatory network and potential signaling pathways on downstream targets is needed in the future to elucidate the pro-tumorigenic role and molecular mechanisms of FANCD2 in PM and develop new targets.

Several limitations of this study should be acknowledged. Because TCGA-MESO lacks matched normal pleural mesothelial tissues, non-tumor lung samples were used as an exploratory comparator. Although the direction of *FANCD2* dysregulation was supported by independent GEO cohorts and by paired clinical specimens, potential tissue-of-origin bias in the TCGA comparison cannot be completely excluded. The TCGA-MESO clinical dataset did not provide complete information on asbestos exposure, occupational history, smoking status, performance status, treatment modality, comorbidities, or all relevant molecular alterations, including *BAP1*, *CDKN2A*, *NF2*, *MTAP*, and *TP53*. Consequently, these variables could not be comprehensively incorporated into the multivariable analysis, and residual clinical and molecular confounding cannot be excluded. The observed association between *FANCD2* expression and survival should therefore be validated in larger cohorts with detailed exposure, treatment, histological, and genomic annotations. In addition, the survival prognostic analysis in this study is exploratory, restricted by the small sample size of the TCGA-MESO cohort and the absence of an independent survival-validation cohort. We verified the proportional hazards assumption *via* Schoenfeld residuals to alleviate model instability, but further verification in large clinical datasets is still necessary. The clinical validation cohort consisted of only ten paired PM and adjacent non-tumor specimens from a single institution. These samples provide preliminary validation of *FANCD2* expression at the mRNA and protein levels but are insufficient to establish diagnostic accuracy or prognostic utility. Multicenter studies with larger sample sizes and complete longitudinal follow-up are required. Furthermore, because proliferation was not experimentally controlled during the wound-healing assay, and FANCD2 knockdown itself reduced proliferation and increased apoptosis, the observed reduction in scratch closure cannot be attributed specifically to impaired migration. Accordingly, the wound-healing data are interpreted only as evidence of reduced wound-closure capacity. A migration-specific assay with appropriate control of proliferation and cell viability will be required to determine whether FANCD2 directly influences PM-cell motility. The functional experiments were conducted in NCI-H2052 cells using two independent *FANCD2*-targeting siRNAs. Therefore, the observed phenotypes represent proof-of-concept findings in this cellular model and should not be generalized to all histological or molecular subtypes of PM without validation in additional cell lines. Future studies should include larger multicenter clinical cohorts with comprehensive clinical information and longitudinal survival data to evaluate the potential diagnostic and prognostic relevance of FANCD2 in PM. We will also continue to explore the biological functions of FANCD2 in PM and its potential molecular mechanisms, providing new potential targets for future PM treatment.

## Data Availability

The datasets presented in this study can be found in online repositories. The names of the repository/repositories and accession number(s) can be found in the article/Supplementary Material. Raw data derived from human specimens cannot be made publicly available due to ethical restrictions and patient privacy protection. De-identified data can be obtained from the corresponding author upon reasonable request.
