# Supplementary material for: FANCD2 knockdown suppresses malignant phenotypes and is associated with caspase/PARP-mediated apoptosis in NCI-H2052 pleural mesothelioma cells
Source: Front Genet. 2026 Sep 23;17:1926600. doi: 10.3389/fgene.2026.1926600 (PMC13641737; doi:10.3389/fgene.2026.1926600)
Supplement: Supplementary file 1 [file Table1.docx]

**Supplementary TABLE 1. Sample source and number cases**

| Database | Normal tissues (n) | PM tissues (n) |
| --- | --- | --- |
| TCGA | 108 | 86 |
| GEO GSE12345 | 4 | 9 |
| GEO GSE42977 | 9 | 39 |
| GEO GSE51024 | 41 | 55 |

PM: pleural mesothelioma; TCGA: The Cancer Genome Atlas; GEO: Gene Expression Omnibus
