# Supplementary material for: FANCD2 knockdown suppresses malignant phenotypes and is associated with caspase/PARP-mediated apoptosis in NCI-H2052 pleural mesothelioma cells
Source: Front Genet. 2026 Sep 23;17:1926600. doi: 10.3389/fgene.2026.1926600 (PMC13641737; doi:10.3389/fgene.2026.1926600)
Supplement: Supplementary file 2 [file Table2.docx]

**Supplementary TABLE 2. qRT-PCR primer sequences and siRNA sequences**

| Primer name | Sequences |
| --- | --- |
| homo-FANCD2 F | 5′-ACATACCTCGACTCATTGTCAGT‐3′ |
| homo-FANCD2 R | 5′-TCGGAGGCTTGAAAGGACATC‐3′ |
| homo-β-actin F | 5′-CATGTACGTTGCTATCCAGGC‐3′ |
| homo-β-actin R | 5′-CTCCTTAATGTCACGCACGAT‐3′ |
| homo-si-FANCD2-1 | 5'-CAGAGUUUGCUUCACUCUCUAdTdT-3' |
| homo-si-FANCD2-2 | 5'-AACAGCCAUGGAUACACUUGAdTdT-3' |
| homo-si-NC | 5'-UCGAAGUAUUCCGCGUACGdTdT-3' |

FANCD2：Fanconi anemia complementation group D2; NC：negative control
