# Supplementary material for: FANCD2 knockdown suppresses malignant phenotypes and is associated with caspase/PARP-mediated apoptosis in NCI-H2052 pleural mesothelioma cells
Source: Front Genet. 2026 Sep 23;17:1926600. doi: 10.3389/fgene.2026.1926600 (PMC13641737; doi:10.3389/fgene.2026.1926600)
Supplement: Supplementary file 3 [file Table3.docx]

**Supplementary TABLE 3. Antibodies information**

| Target Protein | Host Species | Clonality | Application (Dilution) | Source | Identifier |
| --- | --- | --- | --- | --- | --- |
| FANCD2 | Rabbit/IgG | Recombinant monoclonal antibody (D5L5X) | WB (1:1000) | Cell Signaling, USA | Cat#16323S |
| CDK4 | Rabbit/IgG | Recombinant monoclonal antibody (244-257 aa, 3D Epitope) | WB (1:1000-1:4000) | Proteintech, China | Cat#83994-3-RR |
| CDK6 | Rabbit/IgG | Recombinant monoclonal antibody (304-317 aa, 3D Epitope) | WB (1:2000-1:10000) | Proteintech, China | Cat#84200-1-RR |
| Cyclin D1 | Rabbit/IgG | Recombinant monoclonal antibody (286-295 aa, 3D Epitope) | WB (1:5000-1:50000) | Proteintech, China | Cat# 82681-1-RR |
| Cleaved-Caspase 3 | Rabbit/IgG | Recombinant monoclonal 163-175 aa, 3D Epitope) | WB (1:1000-1:4000) | Proteintech, China | Cat#82707-13-RR |
| Cleaved-Caspase 8 | Rabbit/IgG | Recombinant monoclonal 360-374 aa, 3D Epitope) | WB (1:1000-1:4000) | Proteintech, China | Cat#85171-3-RR |
| Cleaved-Caspase 9 | Rabbit/IgG | Recombinant monoclonal antibody (D2D4) | WB (1:1000) | Cell Signaling, USA | Cat#7237S |
| Cleaved-PARP1 | Rabbit/IgG | Recombinant monoclonal 730-743 aa, 3D Epitope) | WB (1:1000-1:4000) | Proteintech, China | Cat#80174-1-RR |
| GAPDH | Rabbit/IgG | Recombinant monoclonal antibody (244-257 aa, 3D Epitope) | WB (1:5000-1:50000) | Proteintech, China | Cat#81640-5-RR |
